# Inulin diet uncovers complex diet-microbiota-immune cell interactions remodeling the gut epithelium

**DOI:** 10.1186/s40168-023-01520-2

**Published:** 2023-04-26

**Authors:** Renan Oliveira Corrêa, Pollyana Ribeiro Castro, José Luís Fachi, Vinícius Dias Nirello, Salma El-Sahhar, Shinya Imada, Gabriel Vasconcelos Pereira, Laís Passariello Pral, Nathália Vitoria Pereira Araújo, Mariane Font Fernandes, Valquíria Aparecida Matheus, Jaqueline de Souza Felipe, Arilson Bernardo dos Santos Pereira Gomes, Sarah de Oliveira, Vinícius de Rezende Rodovalho, Samantha Roberta Machado de Oliveira, Helder Carvalho de Assis, Sergio Costa Oliveira, Flaviano Dos Santos Martins, Eric Martens, Marco Colonna, Patrick Varga-Weisz, Marco Aurélio Ramirez Vinolo

**Affiliations:** 1 Laboratory of Immunoinflammation, Department of Genetics, Evolution, Microbiology, and Immunology, Institute of Biology, University of Campinas, Campinas, SP 13083-862 Brazil; 2 Koch Institute for Integrative Cancer Research at MIT, Cambridge, MA 02139 USA; 3 Department of Pathology and Immunology, Washington University School of Medicine, Saint Louis, MO 63110 USA; 4 International Laboratory for Microbiome Host Epigenetics, Department of Genetics, Evolution, Microbiology, and Immunology, Institute of Biology, University of Campinas, Campinas, SP 13083-862 Brazil; 5 School of Life Sciences, University of Essex, Colchester, CO4 3SQ UK; 6 Department of Gastroenterological and Transplant Surgery, Graduate School of Biomedical and Health Sciences, Hiroshima University, Hiroshima, 734-8551 Japan; 7 University of Michigan Medical School, Ann Arbor, MI 48109 USA; 8 Laboratory of Biotherapeutics Agents, Department of Microbiology, Institute of Biological Sciences, Federal University of Minas Gerais, Belo Horizonte, MG 31270-901 Brazil; 9 Department of Biochemistry and Immunology, Institute of Biological Sciences, Federal University of Minas Gerais, Belo Horizonte, MG 31270-901 Brazil; 10 Department of Immunology, Institute of Biomedical Sciences, University of São Paulo, São Paulo, SP 05508-000 Brazil; 11 São Paulo Excellence Chair, Department of Genetics, Evolution, Microbiology, and Immunology, Institute of Biology, University of Campinas, Campinas, SP 13083-862 Brazil; 12 Experimental Medicine Research Cluster, Campinas, SP 13083-862 Brazil; 13 Obesity and Comorbidities Research Center (OCRC), University of Campinas, Campinas, SP 13083-864 Brazil

**Keywords:** *Bacteroidales*, Epithelial remodeling, Gut homeostasis, High-fiber diet, IL-22, Intestinal stem cells, γδ T cells

## Abstract

**Background:**

The continuous proliferation of intestinal stem cells followed by their tightly regulated differentiation to epithelial cells is essential for the maintenance of the gut epithelial barrier and its functions. How these processes are tuned by diet and gut microbiome is an important, but poorly understood question. Dietary soluble fibers, such as inulin, are known for their ability to impact the gut bacterial community and gut epithelium, and their consumption has been usually associated with health improvement in mice and humans. In this study, we tested the hypothesis that inulin consumption modifies the composition of colonic bacteria and this impacts intestinal stem cells functions, thus affecting the epithelial structure.

**Methods:**

Mice were fed with a diet containing 5% of the insoluble fiber cellulose or the same diet enriched with an additional 10% of inulin. Using a combination of histochemistry, host cell transcriptomics, 16S microbiome analysis, germ-free, gnotobiotic, and genetically modified mouse models, we analyzed the impact of inulin intake on the colonic epithelium, intestinal bacteria, and the local immune compartment.

**Results:**

We show that the consumption of inulin diet alters the colon epithelium by increasing the proliferation of intestinal stem cells, leading to deeper crypts and longer colons. This effect was dependent on the inulin-altered gut microbiota, as no modulations were observed in animals deprived of microbiota, nor in mice fed cellulose-enriched diets. We also describe the pivotal role of γδ T lymphocytes and IL-22 in this microenvironment, as the inulin diet failed to induce epithelium remodeling in mice lacking this T cell population or cytokine, highlighting their importance in the diet-microbiota-epithelium-immune system crosstalk.

**Conclusion:**

This study indicates that the intake of inulin affects the activity of intestinal stem cells and drives a homeostatic remodeling of the colon epithelium, an effect that requires the gut microbiota, γδ T cells, and the presence of IL-22. Our study indicates complex cross kingdom and cross cell type interactions involved in the adaptation of the colon epithelium to the luminal environment in steady state.

Video Abstract

**Supplementary Information:**

The online version contains supplementary material available at 10.1186/s40168-023-01520-2.

## Background

The gut epithelium has the highest cellular turnover rate in adult mammals, which ensures that this large mucosal surface constantly regenerates to fulfill its barrier functions [1]. This dynamic structure made of a single layer of cells is built from crypt-villus units (with villi structures absent in the large intestine). Intestinal stem cells (ISCs) located at the bottom of the crypts proliferate and differentiate into all mature cell types found in the epithelium: enterocytes and M cells (absorptive lineage), Paneth cells, goblet cells, Tuft cells, and enteroendocrine cells (secretory lineage). Due to ISCs continuous proliferation, epithelial cells are in a constant upward movement, an important characteristic to assure tissue homeostasis [2]. Additionally, the intestinal epithelium is equipped with a protective mucus layer produced by goblet cells. This layer acts as a physical barrier between the epithelium and the digestive contents of the gut. In the small intestine, the barrier is a single layer of mucus, while in the colon, there are two layers—an inner that adheres strongly to the epithelium and an outer layer that is loosely attached and can be easily removed [3]. Protective molecules are found embedded in the mucus layer, such as secretory IgA and antimicrobial peptides, the latter secreted by Paneth cells [3]. Gut protective barrier is also reinforced by the presence of immune cells, which can be organized in lymphoid tissues or be dispersed along the lamina propria and within the epithelium itself [3]. Thus, it is crucial to understand how distinct internal and external factors interact with the ISCs as any perturbation in this tightly regulated system can lead to dysfunction of this mucosal barrier and development of several pathologies [4].

Diet is an important environmental factor that strongly impacts the activity of ISCs and, therefore, the functionality of the gut, with most studies focusing on the caloric intake and energy metabolism properties of distinct dietary interventions to the intestinal stem cell niche [5]. However, diet can also indirectly affect the ISCs responses, epithelial architecture, and intestinal inflammatory responses by its ability to modulate the gut microbial composition in the host, although the precise mechanisms behind such complex interactions are still not fully understood [6, 7]. In this context, dietary fibers are the main energy source of the colonic microbiota, and in this way, they are critical food components shaping the gut microbiome. Dietary fibers are divided into two groups: (i) insoluble fibers, which are, in general, less processed during digestion and contribute more to adding bulk to fecal mass and to improving intestinal motility, and (ii) soluble fibers, fermentable carbohydrates that are source of several important microbiota-derived metabolites such as short-chain fatty acids (SCFAs), bile acids, polyamines, ions, phenols and vitamins, all with distinct actions in the host cells [8–10]. Soluble fibers such as inulin, pectin, beta-glucans, fructo-oligosaccharides, galacto-oligosaccharides, and resistant starch are found naturally in many foods, such as fruits, vegetables, whole grains, and legumes. For example, inulin is found in roots such as from chicory and Jerusalem artichokes, while pectin is found in apples and citrus fruits. The literature on the effects of the ingestion of soluble fiber is large, although outcomes vary significantly among studies due to factors including the type of fiber, their concentration, gut microbial structure, and interactions among other dietary components [11].

Nonetheless, several studies have collectively claimed that the consumption of elevated levels of fermentable fibers can confer protection against several intestinal and extraintestinal pathologies when compared to low soluble fiber intake [12–23]. Consumption of dietary pectin in mice, for example, was shown to protect the intestinal stem cell pool from injuries caused by radiation, improving intestinal crypt regeneration and overall survival of animals [24]. However, not all fibers are well tolerated, as sensitivity and detrimental effects have been reported mostly by patients with inflammatory bowel diseases (IBD) [25, 26]. A recent study indicated that such negative effects can, in part, be explained by the lack of microbiota fermentative activities in some of IBD patients, resulting in the presence of unfermented β-fructan fibers that can activate pro-inflammatory responses in the gut [27]. This places bacterial fermentation as a crucial step to achieve the benefits of fiber consumption. However, as alluded above, soluble fibers have also shown to have direct effects on intestinal epithelial cells [27–30], even possibly through mechanisms that are independent of the gut microbiota [31]. Thus, unraveling microbiota-dependent versus independent roles of soluble fibers is important to understand their mechanisms.

Inulin, which is classified as prebiotic [32], has gained scientific attention among fermentable fibers due to its increasing usage in the food industry and subsequent prevalence in the human diet [33–35]. In vivo studies reported that ingestion of inulin can prevent detrimental phenotypes induced by unbalanced (high-fat/high-sugar) diets, both in humans [36, 37] and mice [18, 38–40], even when administered at very low concentrations [41]. However, deleterious effects of inulin have also been reported in animals facing intestinal inflammatory conditions [42, 43], as well as mice ingesting high doses of this soluble fiber, such as demonstrated by a recent study showing enhanced type 2 inflammation in animals fed with a diet enriched with 26% of inulin [44]. In vitro and in vivo studies indicated that inulin improves the epithelial barrier, an effect associated with increased expression of tight and adherens junction genes [28, 29, 39, 45–48]. Interestingly, inulin originated from the same plant source (chicory root) but with distinct levels of purity and polymerization presented different effects on intestinal barrier, indicating that these physicochemical aspects are relevant for the biological effects of this fiber [15, 27]. Modulation of inflammatory cytokines [45, 46] and increased production of mucus [49] and defensins [18, 39, 45, 46] have also been reported in the literature, which collectively demonstrates that inulin has significant effects on different aspects of the intestinal epithelium. Moreover, inulin has also shown to regulate epithelial proliferation and differentiation [18]. In this regard, understanding the impact of inulin in the host has become an important research focus, especially considering the lack of knowledge of its effects in the activity of ISCs. We hypothesized that inulin consumption modifies the composition of colonic bacteria and this impacts intestinal stem cells functions, thus affecting the epithelial structure. We found that intake of inulin drives an epithelial remodeling in the murine colon, associated with increased proliferation of ISCs and differentiation of goblet cells compared to a control diet enriched only with insoluble fiber. Such effects required the gut microbiota, the action of γδ T lymphocytes and the presence of IL-22.

## Methods

### Mice

Adult C57BL/6/J mice were purchased from the Multidisciplinary Centre for Biological Investigation (CEMIB-UNICAMP), Campinas-SP, Brazil. *Ffar2* deficient mice (*Ffar2*^*−/−*^) were generated as described previously [50]. TCRδ^−/−^ mice and Rag-1^−/−^ mice were purchased from the Special Mouse Breeding Center (CCCE) of the University of São Paulo at Ribeirão Preto-SP. All animals were maintained in the animal facility of the Department of Genetics, Evolution, Microbiology, and Immunology of the Institute of Biology, University of Campinas. *Il22*^*−/−*^ deficient mice were provided by S.C.O. and maintained in the Department of Microbiology, Institute of Biological Science of the Federal University of Minas Gerais, as well as both germ-free and specific-pathogen free animals of Swiss background. Aerobic and anaerobic bacterial cultures of fecal samples, together with PCR tests on breeding colonies were regularly performed to validate the germ-free status of the mice. *Lgr5-EGFP-IRES-CreERT*2 (B6.129P2-Lgr5^tm1(cre/ERT2)Cle^/J) were obtained from the Jackson Laboratory. *Lgr5-IRES-CreERT2* mice [51] were crossed to tdTomato^LSL^ mice (Jackson Laboratory, #007,909) to generate *Lgr5-CreERT2; tdTomato*^*LSL*^ mice. These animals were maintained in the husbandry care of the Department of Comparative Medicine in the Koch Institute for Integrative Cancer Research. Gnotobiotic SM13 mice [52] were provided by E.C.M. Rorγt-Cre Ahr floxed mice and TCRβ^−/−^ mice were provided by M.C. and maintained in specific pathogen-free facilities at Washington University in Saint Louis. All strains were maintained in a C57BL/6 background and all procedures were carried out using 6–8-week-old mice, unless otherwise specified. All strains were kept in regular filter-top cages with free access to sterile water and food and with no more than 5 mice per cage. EdU was prepared at 10 mM in PBS and 250 µL was injected intraperitoneally (i.p.) per animal. BrdU was prepared at 10 mg/mL in PBS, passed through a 0.22 μm filter and injected i.p. at 100 mg/kg. Sunflower seed oil (Spectrum S1929) was used to suspend tamoxifen at a 10 mg/mL concentration, which was administered (250 µL per 25 g of body weight) i.p. according to the time points described in the main text and figure legends. For treatment with antibiotics, C57BL/6 mice received a mix of 1 g/L ampicillin, 1 g/L neomycin, 1 g/L metronidazole and 0.5 g/L vancomycin (as described by [53]) in the drinking water for 30 days, while being fed with the inulin-enriched diet.

### Dietary approaches

Diets were prepared in the Laboratory of Cereals, Roots, and Tubers (FEA/UNICAMP) following the recommendations of the American Institute of Nutrition (AIN-93) [54]. The control diet was composed of 5% cellulose (insoluble fiber), and the inulin-enriched diet was composed of 5% cellulose + 10% inulin (soluble fiber). High-fiber diets enriched with 15% cellulose, 5% cellulose + 2% inulin, 5% cellulose + 5% inulin, 5% cellulose + 10% pectin or 5% cellulose + 5% fructooligosaccharide (FOS) were prepared similarly. The detailed composition of all diets is in Table S1. Similar control or 10% inulin diets were purchased from Research Diets Inc. (D10012-M and D19071901, respectively) for the experiments performed in the USA. Standard animal facility chow (Nuvilab CR-1) (hereby named as Chow diet) contained 20% of insoluble fiber and 0.2% of soluble fiber according to the manufacturers (MA-046 Official Methods of Analysis of the Association of Analytical Chemists.—Method 991.43 – 18th ed.) was also used. Mice were provided with the diets for 30 days, unless otherwise specified. Once euthanized, the intestine was harvested and measured, with this value normalized by the weight of each correspondent mouse.

### Weight gain and food consumption

Each mouse had its body weight measured before starting on the different diets, as well as once a week until euthanasia. A measured amount of food was placed in a cage and weighted twice a week before adding more to the remaining portion. After 30 days, the remaining food was also measured and subtracted from the total amount added in the cage. Then, the total amount of consumed food was divided by the number of mice in the cage, and by the number of days in the diets. This allowed estimates of the amount of food consumed by each animal per day.

### Histological analysis

Mouse small intestines and colons were harvested and fixed in 4% formalin, embedded in paraffin, and sectioned for staining with Hematoxylin–Eosin (H&E) or Alcian blue-periodic Acid-Shiff (AB/PAS) solutions. For immunohistochemistry, Borg Decloaker RTU solution (Biocare Medical) was used for antigen retrieval in a pressurized Decloaking Chamber (Biocare Medical) for 5 min. Antibodies used: rabbit anti-Ki-67 (1:4000, Cell Signaling 122,025), rat anti-BrdU (1:2000, Abcam 6326), rabbit polyclonal anti-RFP (1:500, Rockland 600–401-379), biotin-conjugated secondary donkey anti-rabbit or anti-rat (Jackson ImmunoResearch). For visualization, Vectastain Elite ABC immunoperoxidase detection kit (Vector Labs PK-6101) and Dako Liquid DAB + Substrate (Dako) were used. All antibody incubations were performed with Common SignalStain (R) Antibody Diluent (Cell Signaling 8112L). For EdU histological analysis, Click-iT reaction was performed on paraffin slides following the manufacturer's protocol (Thermo Fisher). For all histological analysis, individual crypts were analyzed, with an average of 150 crypts per mouse.

### RNA in situ hybridization

The specificity of the Mm-*Smoc2* probe (ACD RNAscope®) was assessed using DIG RNA labeling mix (Roche) according to the manufacturer’s instructions as described previously [55]. The probe corresponds to expressed sequence tags from Open Biosystems. The Advanced Cell Diagnostics RNAscope 2.0 HD Detection Kit was used to perform the single molecule ISH.

### Analysis of the transcriptional profile of the intestinal epithelial cells

To extract the intestinal epithelial cells (IECs), the colon was harvested, cut longitudinally and washed 3 × with ice-cold PBS, followed by 3 washes with Hank’s balanced salt solution (HBSS) containing 5% fetal bovine serum (FBS). The colon was cut into smaller pieces of 0.5 cm and incubated in HBSS containing 2 mM EDTA for 1 h at 37 °C under agitation. The solution was filtered with 100 µm and 70 µm cell strainers and centrifuged (300 × g, 10 min, 4 °C). The cell pellet was washed with ice-cold HBSS to remove EDTA and centrifuged again. The number of cells was determined using a Neubauer’s chamber and the cell pellet was frozen in liquid nitrogen and kept at -80 °C. Total RNA was extracted from 2 million IECs using the PureLink™ RNA Mini Kit (ThermoFisher) according to the manufacturer’s instructions. RNA quantification was measured using a Nanodrop, and the RNA integrity using the Bioanalyzer RNA 6000 Pico chip (Agilent). Library preparation for RNA-Seq was performed with 500 ng of RNA using NEBNext® UltraTM Directional RNA Library Prep Kit for Illumina and the NEBNext® Poly(A) mRNA magnetic isolation module. Illumina TruSeq adaptors were used, and the amplification of libraries was performed with KAPA PCR Amplification kit (KAPA, Cat. KK2501) with 14 cycles. Libraries were then sequenced in the HiSeq2500 (Illumina) according to the manufacturer’s instructions. Raw data were analyzed using SeqMonk software (Babraham Institute Bioinformatics – Cambridge, UK) and gene ontology was determined using DAVID 2021 [56].

### Crypt isolation and single cell dissociation

To obtain single IECs, the colon was harvested, opened longitudinally, washed in cold PBS to remove feces, and cut into smaller pieces, which were put into microtubes with HBSS-10 mM EDTA-1% P/S (penicillin/streptomycin). After incubation (1000 rpm agitation, 20 min, 37 °C), the tissue pieces were removed and the tubes centrifuged (300 × g, 5 min, 4 °C). The pellet was washed with cold HBSS-0.04% -1% P/S to remove EDTA. After centrifuging again, HBSS-0.04% BSA-1% P/S and 10 mM dithiothreitol (DTT) were added to remove mucus (room temperature [RT], 10 min). Cold PBS-0.04% BSA was then added, and the suspension was filtered through a 70 µm cell strainer. After centrifugation, TrypLE Express enzyme (Thermo Fisher) + DNAse 50 µg/mL was added and gently pipetted up and down to dissociate the pellet. After incubation (1000 rpm mix, 5 min, 37 °C), HBSS-0.04% BSA-1% P/S was added to stop the reaction. Then, cold PBS-0.04% BSA was added, the suspension was filtered (40 µm cell strainer), centrifuged and resuspended in cold PBS-0.04% BSA.

### Droplet-based scRNA-Seq 

The single-cell suspensions were immediately loaded onto the Chromium Next GEM Chip G (10X Genomics), according to the manufacturer's protocol CG000388. Quality control of libraries was performed on the Bioanalyzer high-sensitivity chip. The libraries were sequenced on an Illumina HiSeq, paired end, with a read length of 150 bp.

### scRNA-Seq quantification and statistical analyses

FASTQ reads were processed using Cell Ranger v6.0.1 (10X Genomics). Reads were aligned to the mm10 mice transcriptome and cell barcodes and UMI (Unique Molecular Identifier) were extracted. The generated gene expression matrices were filtered to remove data from poor-quality cells. First, we estimated cell-free mRNA contamination and removed it with the SoupX library, comparing the raw and filtered matrices obtained with cellranger [57]. After that, genes that were expressed in less than 3 cells were not considered. We removed cells possessing fewer than 200 detected genes using Seurat v.4.1.0 [58] and performed doublet removal using the scDblFinder library [59]. In addition, we removed cells with a percentage of mitochondrial genes greater than 25%. The variability between cells derived from technical and biological effects was corrected using the IntegrateData function in Seurat.

After dimensionality reduction of integrated data, the cell-type identification was performed by graph clustering at a resolution of 1.2 with 30 principal components (PCs). We used the function FindAllMarkers to determine the marker genes for each cluster. Using Receiver Operating Curve (ROC) analysis, we encountered clusters containing highly discriminative marker genes in mice, or orthologs in humans. We made annotations by manual analysis of the main marker genes in the clusters.

We first looked at markers to differentiate epithelial from immune and stromal cells (described by [60]) (Table S2). After confirming epithelial cells, we classified two levels differentiating between absorptive, secretory, and proliferative, using known markers also described by Smilie et al. [60]. Then, we classified cells into Enterocytes, Immature Enterocytes, Goblet cells, Cycling Transit-Amplifying (Cycling TA), or Stem cells, in addition to well-characterized but rarer cell types such as Tuft cells and Enteroendocrine cells. For this, we used a set of known markers [60, 61] or markers present in databases as PanglaoDB [62] and CellMarker [63]. Clusters that did not present markers for any cell type of interest or contained low-quality cells were removed.

The Speckle v0.0.3 [64] was used to estimate the relative differences in cell-type proportions between conditions. A t-test was used to calculate p-values and Benjamini–Hochberg false discovery rates were calculated to account for multiple testing of cell types/clusters. The cell-type differential expression analysis was performed using Model-based Analysis of Single-cell Transcriptomics (MAST) [65]. Each cell type grouped by condition was tested by the Seurat differential gene expression testing framework (corrected p-value cut-off < 0.05). We calculated the cell cycle score using the CellCycleScoring function with a set of genes for S and G2/M phases [66]. In addition to the cell cycle score, this library also predicts the cell cycle phase. The p-value of the relative difference in cell cycle proportion was calculated using Fisher's Exact test.

### Organoid clonogenicity assay

Distal colon was harvested, washed with cold PBS, opened longitudinally and then incubated at 37 °C with shaking at 1000 rpm with PBS + EDTA (10 mM) for 30 min. Tissues were then transferred to PBS and isolated crypts were counted and seeded in Matrigel™ (Corning 356,231 growth factor reduced) at 200 crypts per well (96-well plate) and cultured in medium as follows: Advanced DMEM (Gibco) supplemented with epidermal growth factor (EGF; 40 ng/mL; R&D), Noggin 200 ng/mL (Peprotech), R-spondin 500 ng/mL (R&D or Sino Biological), N-acetyl-L-cysteine (1 μM; Sigma-Aldrich), B27 (1X; Life Technologies), Chiron 10 μM (Stemgent), and Y-27632 dihydrochloride monohydrate (20 ng/mL; Sigma-Aldrich). Droplets (7 μL) of Matrigel™ containing the crypts were plated onto a flat bottom 96-well plate (Corning 3548), which was incubated for 20 min at 37 °C to allow Matrigel™ solidification. Crypt culture medium (150 μL) was then overlaid onto the Matrigel™ and kept at 37 °C in fully humidified chambers containing 5% CO_2_. Clonogenicity (colony-forming efficiency) was determined by assessing organoid formation 5 days after seeding.

### 16S rRNA gene processing and analysis

Microbial DNA was extracted from colon luminal content samples using the PureLink™ Microbiome DNA Purification kit (Thermo Fisher). 16S rRNA V3-V4 variable regions were amplified and sequenced by BGI Hong Kong as 250 bp paired-end reads (Illumina) and were analyzed using the 16S rDNA-amplicon pipeline. Briefly, low-quality and adaptor-polluted reads were removed prior to paired-end merging. The de-multiplexed sequences were processed using the DADA2 pipeline [67] after passing FastQC quality checks. Reverse reads failed the checks, thus only forward reads were used. The parameters used were slightly modified from the DADA2 original workflow to account for the quality trimming required. The fastq files were then filtered based on the error rates and quality scores generated by the DADA2 algorithm. Following this, the reads were subjected to noise removal and summarized into amplicon sequence variants (ASVs), then filtered for chimeric sequences. Taxonomy was assigned using the RDP Classifier training set 18 [68]. The *phyloseq* R package was used for downstream analysis, including the heatmap plots. Shannon’s index, Chao1 and observed richness were calculated to assess taxa diversity and evenness. To explore the variability among sample groups, UniFrac distances between the samples were calculated and visualized using Principal Coordinates Analysis (PCoA). The statistical differences between microbial communities were tested using DESeq2 [69]. LEfSe analysis was used to determine the ASVs that were statistically different among the two experimental groups [70]. A linear discriminant analysis (LDA) threshold of 2 was used and a significance alpha value of 0.05 was set for both the Kruskal–Wallis and Wilcoxon tests. The top 20 ASVs were sorted by LDA scores and plotted to show statistically significant differentially abundant taxa.

### Fecal microbiota transplantation to germ-free mice

Swiss germ-free (GF) mice received fecal microbiota from Swiss specific-pathogen free (SPF) mice previously fed with control or inulin diet for 30 days. The microbiota transfer was performed first by co-housing, where GF animals were kept for 48 h in the same cages previously occupied by the SPF donor animals. Both feces and colonic luminal content were collected and diluted in PBS (1 g of feces to 10 mL of PBS). The fecal content was homogenized, followed by sedimentation for 10 min. The suspension (100 µL) was then given to the GF recipient mice in a single dose by oral gavage. Next, the recipient animals were kept in positive-pressure individually ventilated cages for 21 days for bacterial colonization, being fed with standard chow, control, or inulin diets, and then euthanized for tissue harvesting.

### Gnotobiotic SM13 mice

Experiments using a synthetic microbiota were performed as previously described [52]. Adult C57BL/6 germ-free parent mice were colonized with a consortium of 14 bacteria species ([52] Table S3). Littermates generated from them were weaned at 21 days old and exposed to the diets (Control or Inulin diet). After 30 days, mice were euthanized and their bacterial composition was assessed by 16S rRNA sequencing, as well as the intestinal epithelial responses as described above. All bacterial species from the consortium were transmitted, with exception of *Faecalibacterium prausnitzii*. However, the presence of this species was not necessary for the phenotype induced by inulin intake. The experiments with these mice were performed in positive-pressure individually ventilated cages containing not more than 5 mice from the same breeders.

### Measurement of short-chain fatty acids

Colonic luminal content (30 mg) was homogenized in 100 µL milliQ water and mixed with 10 mg citric acid, 20 mg sodium chlorite, 40 µL 1 M hydrochloric acid, 200 µL butanol, and 20 µL caprylic acid. Samples were vortexed, 50 µL of the organic phase was collected, and 1 µL injected into the gas chromatograph with a 1:25 split. The chromatograph used was the GC-2010 Ultra (Shimadzu Scientific Instruments Inc., Kyoto, Japan) with a Stabilwax fused silica capillary column (Restec Corporation, USA) of 30 m × 0.25 mm of internal diameter coated with a 0.25 µm thick layer of polyethylene glycol. High quality pure helium was used as a carrier gas (1 mL/min constant flow). The temperature gradient started at 100 °C, with a 2-min hold, increased to 110 °C (15 °C/minute of rate with a 3-min hold), increased again to 140 °C (10 °C/minute), and finally it was increased to 230 °C (70 °C/minute of rate with a 2-min hold). The time for each analysis was 11.95 min. Mass conditions were as follows: ionization voltage of 70 eV; ion source temperature of 200 °C; and full scan mode in the mass range 35–500 at 0.2 s/scan. A calibration curve was made ranging from 0.015 to 0.1 mg/mL and the retention times of standards (Volatile Free Acid Mix, code 46,975, Sigma Chemical Co., St. Louis, MO, USA) were used to identify the individual metabolites.

### Quantitative gene expression

The PureLink™ RNA kit (Thermo Fisher) was used to extract total RNA from the isolated cells. The High-Capacity cDNA Reverse Transcription Kit (Applied Biosystems) was used to perform the conversion of RNA to cDNA, and quantitative polymerase chain reaction (qPCR) was performed with the Power SYBR Green PCR Master Mix (Applied Biosystems) and the primers listed in Table S4. Gene expression was quantified by the 2^ΔΔ^CT method, using β2-microglobulin as a reference gene. The QIAamp DNA Stool Mini Kit (Qiagen) was used to extract total DNA from fecal samples according to the manufacturer’s protocol. For fecal bacterial load quantification, universal primers targeting 16S rRNA were used, as listed in Table S4. A standard curve made of *E. coli* genomic DNA was also used.

### Intestinal lamina propria immune cells isolation

Small intestine and colon were harvested, and mesenteric adipose tissue and Peyer’s patches removed by dissection. The intestines were opened longitudinally, washed with ice-cold HBSS-5% FBS to remove the luminal content, and cut into smaller pieces. Intestinal epithelial cells were first isolated by washing and vortexing the tissues in ice-cold PBS + 5 mM EDTA and collected for gene expression analysis. IELs were isolated using a 40–70% percoll gradient from tissue washes with ice-cold PBS + 5 mM EDTA. Intestinal lamina propria immune cells were then isolated by digestion with 1 mg/mL collagenase IV (Sigma) in complete RPMI for 40 min, at 37 °C, under agitation. Cells were washed and counted using a Neubauer’s chamber, followed by ex vivo stimulation with complete RPMI with IL-1β (10 ng/mL), IL-23 (10 ng/mL) and brefeldin A (1:1000, BD GolgiPlug), for 3 h, in 96 round-bottomed wells polystyrene plates (Corning). After this, cells were labeled with antibodies for FACS analysis (described below).

### Flow cytometry

To exclude non-viable cells, a live/dead viability assay (Brilliant Violet 510) was used. Innate lymphoid cells (ILCs) were identified with a lineage cocktail of R-phycoerythrin-conjugated monoclonal antibodies against CD3, CD11c, CD11b, CD19, Ly6C, Ly6G and TCR, all diluted in FACS-buffer. ILC was defined as Lin- and CD45 + (PE-Cy7). Subtypes of ILC3 were identified with Nkp46 (BV421), CCR6 (BV605) and CD90.2 (BV785) labeling. The lymphocyte populations were identified as CD45 + , CD3 + , CD4 + , TCRγδ, and TCRβ. Fc receptors blockage was done with purified anti-CD16/CD32 (Biolegend) and staining of the surface antigens was performed in the dark, at 4 °C, for 20 min. Foxp3 Staining Buffer Set (eBioscience) with RORγt (Percp-Cy5.5), Gata3 (FITC) and FoxP3 (BV421) monoclonal antibodies was used for cell fixation and intracellular staining following the manufacturer’s instructions. IL-22, IL-17 and Ki67 were stained with monoclonal antibodies after fixation and permeabilization of cultured cells. Samples were analyzed using the FACS-Symphony™ (BD Biosciences) and the FACSDiva™ Software (BD biosciences). All FACS data were analyzed using FlowJo LLC v.10.1 software (Becton Dickinson). Regarding intestinal stem cells analysis, epithelial cells were first isolated from the colon as already described and the GFP + population was identified by FACS.

### Statistical analysis

GraphPad Prism 8.0 software was used for the analyses. All data are presented as violin plots unless otherwise stated in the figure legends. In general, data were analyzed for normal distribution by Kolmogorov–Smirnov normality tests and compared as appropriate by unpaired two-tailed t-test or Mann Whitney U-tests. Details of individual tests are included in the figure legends. Differences were compared by one-way or two-way ANOVA for multiple data sets. In all cases, statistical significance was considered when *p* < 0.05.

## Results

### Ingestion of inulin stimulates cell proliferation in the colon

**Fig. 1:**
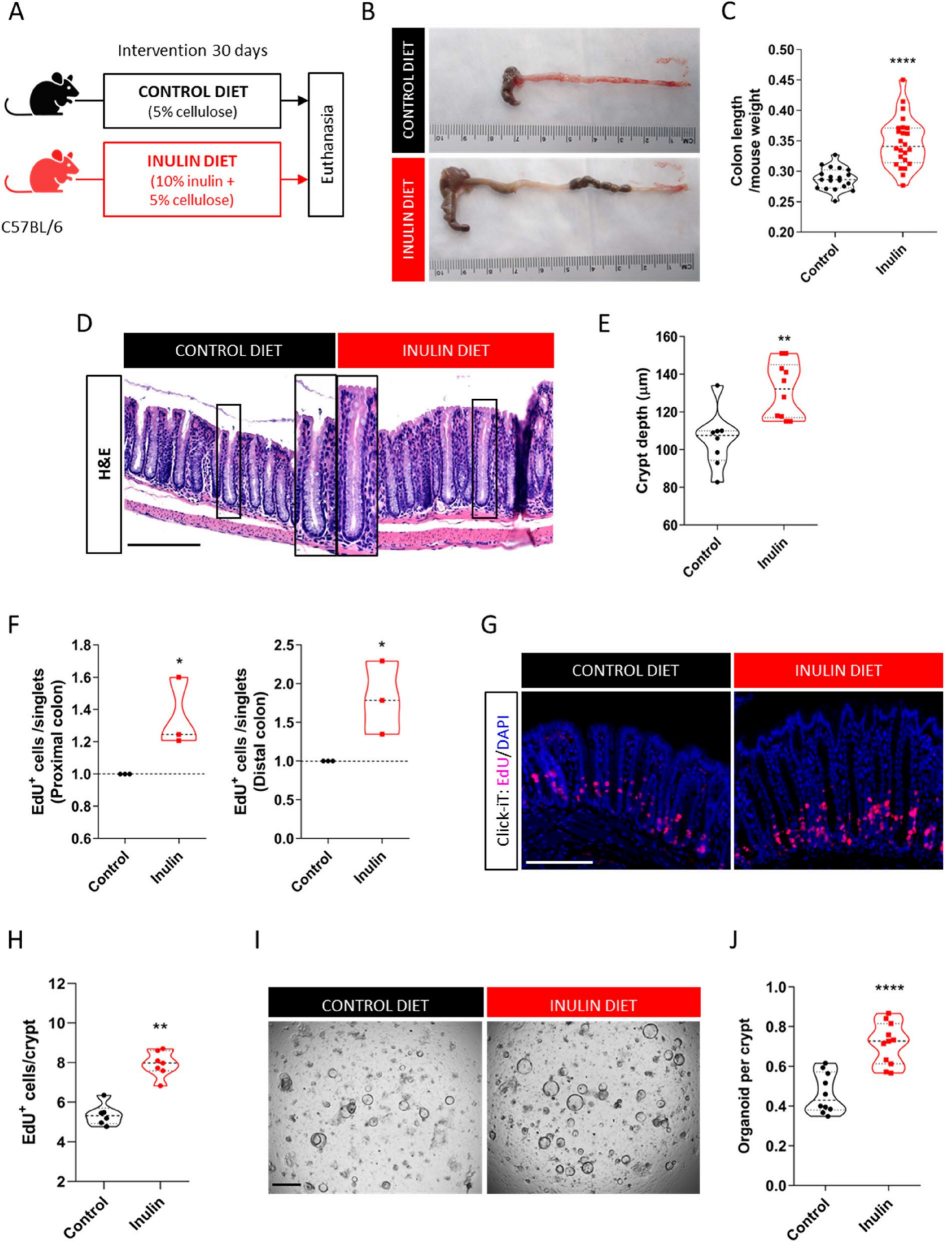
Ingestion of inulin stimulates cell proliferation in the colon. **A** Experimental model scheme with two dietary groups. **B** Representative images of the harvested cecum and colon after 30 days of indicated diet. **C** Quantification of colon length normalized by mice weight (*n* = 19–24). Data pooled from 4 independent experiments. **D** Representative images of colonic epithelium by H&E staining. Scale bars, 100 µm. **E** Measurement of colon crypt depth (*n* = 8–10). Data were pooled from 2 independent experiments. **F** Quantification of the number of EdU-positive cells normalized by the number of acquired singlets by flow cytometry in both proximal and distal regions of the colon (*n* = 3). **G** Visualization of EdU-positive cells in colonic crypts by fluorescence microscopy following EdU Click-iT reaction. Scale bars, 50 µm. **H** Quantification of EdU-positive cells per crypt (*n* = 6–7). Data were pooled from 2 independent experiments and analyzed by Mann–Whitney test. **I** Representative images of crypts-derived colon organoids 5 days in the culture. Scale bars, 1 mm. **J** Quantification of the clonogenicity capacity of colon crypts (*n* = 10–11). Data were pooled from 2 independent experiments. In all graphs, each point represents an individual animal. Unless otherwise stated, results were analyzed by Student’s t-test. *p < 0.05, **p < 0.01, ****p < 0.0001

To identify the effects of inulin ingestion in the colonic epithelium, adult C57BL/6 mice were fed for 30 days with a diet containing 5% of cellulose (AIN-93 M) with or without a 10% inulin supplementation (hereby named Control diet and Inulin diet, respectively) (Fig. 1A). We found that compared to the control diet, the inulin diet significantly increased the length of the cecum and colon (Fig. 1B and C), accompanied by 25% longer colonic crypts in average, both in proximal and distal colon (Fig. 1D and E). No differences were observed regarding body weight, food consumption and colon crypt density between the two diet groups (Fig. S1A, B and C, respectively), indicating that such intestinal enlargement does not occur due to organ distension caused by the presence of larger amounts of food, mucus and/or bloating. Moreover, the effect of the inulin diet was specific for the large intestine, as no differences in crypt depth and villus length were observed throughout the small intestine (duodenum, jejunum, and ileum) (Fig. S1D), suggesting a potential role of bacterial fermentation of inulin that takes place mostly in the colon and not in the small intestine. To understand whether these morphological alterations could be related to modulation of the epithelial proliferative rate, we performed a nucleotide analogue EdU incorporation assay. Flow cytometry analysis revealed significantly more EdU^+^ cells in both proximal and distal colon of mice fed with the inulin diet compared to the control ones (Fig. 1F), a phenotype also confirmed by histological analysis (Fig. 1G and H). This increased cell proliferation was corroborated by BrdU incorporation (Fig. S1E and F) and immunostaining for the proliferative cell marker Ki67 (Fig. S1G and H). We highlight that similar proliferation induction by the inulin diet was observed in both female and male mice, as well as in mice housed in different vivaria, which indicate that this phenotype is robust and not affected by sex or distinct animal facilities. We also performed a clonogenicity assay by seeding colon crypts from mice fed the control or inulin diets in a 3D culture. Consistent with histological results, the freshly isolated crypts revealed the size difference between the two diet groups (Fig. S1I). After 5 days in culture, the number of colon crypts to develop into organoids was on average 35% higher in the inulin diet mice compared to the control group (Fig. 1I and J), showing a higher regenerative capacity of the colon crypts ex vivo.

Next, we aimed to check if these phenotypes were induced due to the presence of inulin, a highly fermentable fiber, or due to the discrepancy of the total fiber content between the two diets (Control diet contains 5% [cellulose only] and Inulin diet contains 15% [cellulose + inulin]). For this, besides the two dietary conditions above, C57BL/6 mice were kept for 30 days on one of the following diets: 15% cellulose diet (matching the total fiber content of the Inulin diet group), chow diet provided by the animal facility (Nuvilab Cr-1 containing 20% insoluble + 0.2% soluble fibers from distinct sources), and diets enriched with lower levels of inulin (2% or 5%, plus 5% cellulose) (Fig. S1J, Table S1). Mice on either chow diet or 15% cellulose shared the same epithelial responses as those from the control group, indicating that this phenotype was not due to a bulking effect of inulin. The results of this experiment also revealed a dose-dependent response of inulin, with lower concentrations already driving the epithelial phenotypes observed with the 10% inulin diet (Fig. S1K and L). Lastly, C57BL/6 mice kept for 30 days on diets enriched with other types of soluble fibers (Fig. S1M) showed that the induction of epithelial proliferation is not exclusive to inulin intake, as similar effects could be achieved by the ingestion of pectin or fructooligosaccharide (FOS) (Fig. S1N). Collectively, these results indicate that the ingestion of highly fermentable fibers, but not of cellulose, impacts the colonic epithelial compartment by enhancing its proliferative rate, an effect that leads to deeper crypts and longer colons in the steady state.

### Ingestion of inulin enhances the proliferative activity of colonic Lgr5^+^ stem cells

**Fig. 2:**
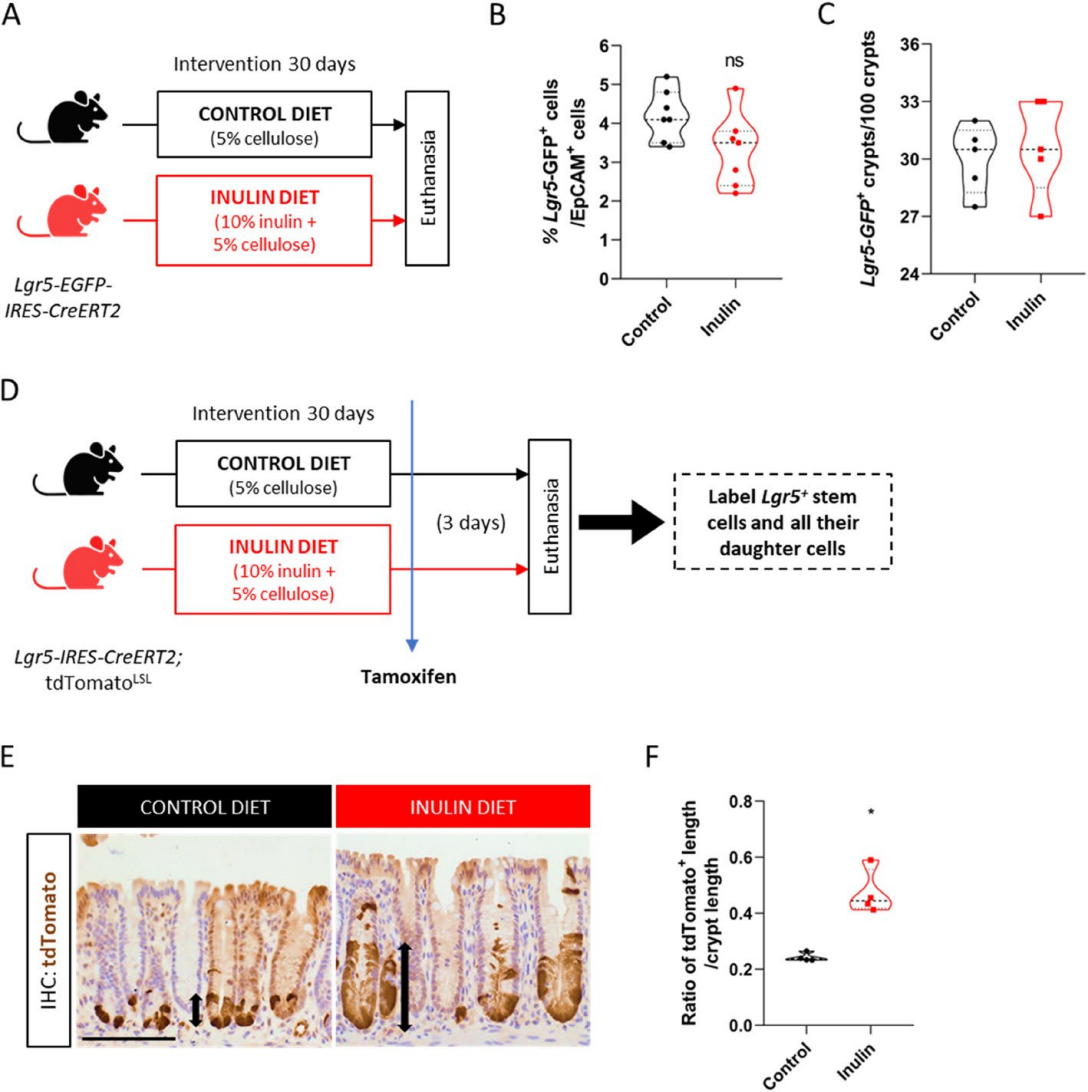
Ingestion of inulin enhances the proliferative activity of colonic Lgr5 + stem cells. **A** Experimental model scheme with reporter mice and two dietary groups. **B** Quantification of the percentage of Lgr5-GFP-positive cells in the colon by flow cytometry (*n* = 7). **C** Quantification of the percentage of Lgr5-GFP-positive crypts in the colon by immunohistochemistry (*n* = 5). **D** Experimental model scheme with lineage-tracer mice and long-term (3 days) tamoxifen injection. **E** Visualization of tdTomato-positive cells in colonic crypts by optical microscopy following staining with anti-tdTomato antibody. Scale bars, 100 µm. **F** Quantification of the ratio of tdTomato-positive length relative to crypt length (*n* = 4). In all graphs, each point represents an individual animal. Results were analyzed by Student’s t-test. *p < 0.05, and ns = not significant

Based on the enhanced epithelial proliferation described above, we next aimed to characterize the impact of the diet enriched with 10% inulin in the colonic stem cell compartment. Given that Lgr5 is a highly specific marker for intestinal stem cell population located at the bottom of the crypts [71], we fed the *Lgr5-EGFP-IRES-CreERT2* ISC reporter mice with either the control diet or inulin diet for 30 days (Fig. 2A). With that, we observed no differences in the number of Lgr5^+^ cells by flow cytometry (Fig. 2B) or Lgr5-labeled crypts by immunohistochemistry (IHC) (Fig. 2C) analyses between the two dietary groups. Smoc2 is another marker that is highly expressed in the Lgr5^+^ ISC population [72]. In this regard, i*n situ* hybridization of *Smoc2* also revealed similar numbers of Smoc2^+^ cells in the colonic crypts between both diets (Fig. S2A and B). We also measured the stem cell pool using the Lgr5-tdTOMATO lineage-tracer mouse. A 24-h pulse of tamoxifen allowed the labeling of the Lgr5^+^ stem cells (Fig. S2C and D) which, again, showed a similar number of tdTomato^+^ cells in the bottom of the crypts (Fig. S2E) and a similar number of Lgr5-tdTomato^+^ labeled crypts in both diets (Fig. S2F). However, when given a 72-h pulse of tamoxifen, which allowed the labeling of the Lgr5^+^ stem cells and all their daughter cells moving upwards into crypts (Fig. 2D), we observed that the inulin diet significantly increased the length of the tdTomato^+^ area in the crypts by approximately two-fold compared to control mice (Fig. 2E and F), revealing enhanced stem cell function to generate more progenies. Collectively, these data show that the increased epithelial proliferation induced by the ingestion of inulin is due to the impact of this soluble fiber in the colonic stem cell compartment by enhancing the proliferative activity of ISCs without altering their number.

### Inulin diet affects distribution and transcriptional profile of colon epithelial cell populations

**Fig. 3:**
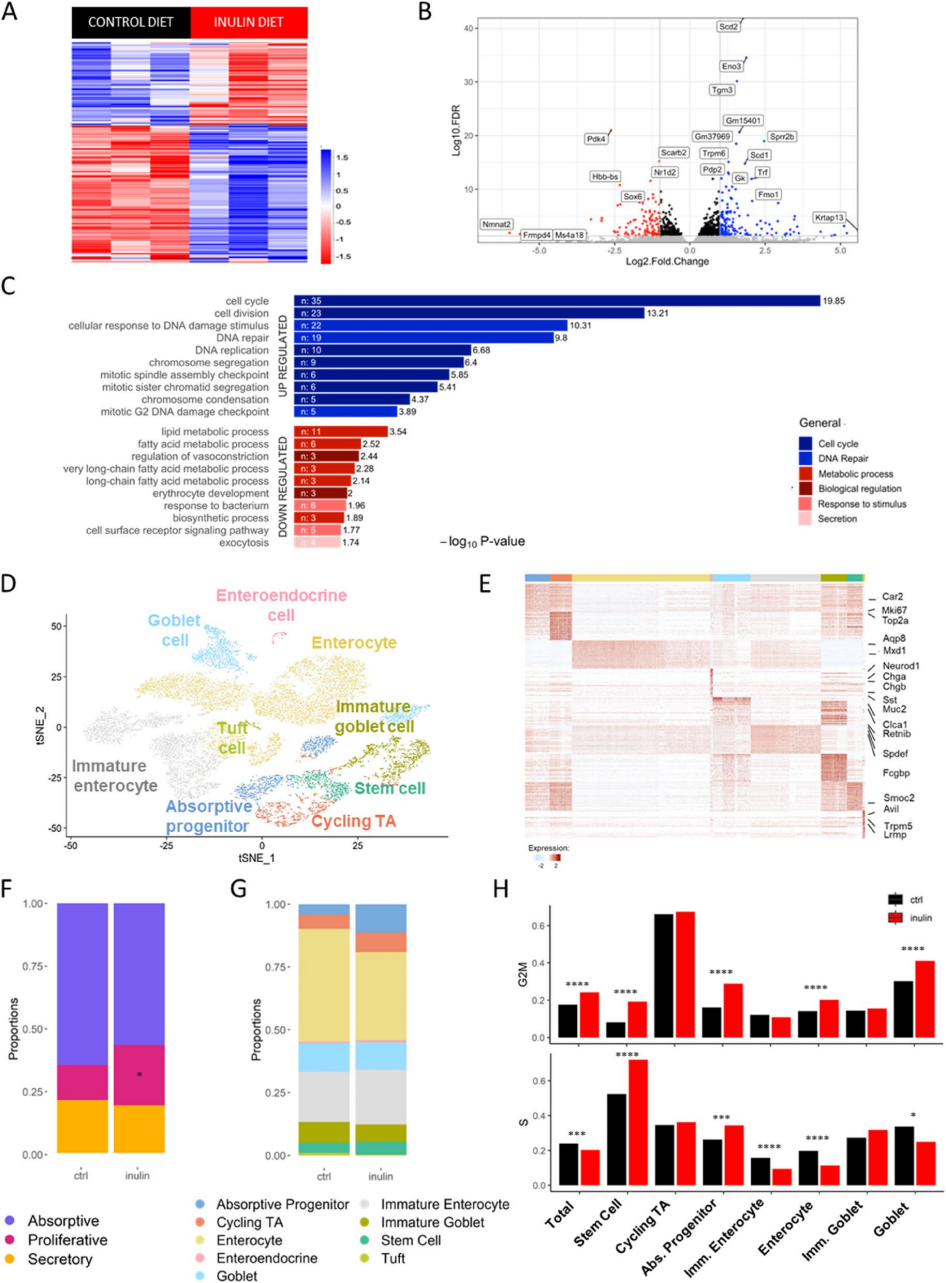
Inulin diet affects distribution and transcriptional profile of colon epithelial cell populations. **A** Heatmap showing up- or downregulated genes in the colon epithelial cells of both dietary groups after transcriptome analysis. DESeq statistical test with significance threshold *p* < 0.05 (*n* = 3). **B** Volcano plot displaying up- (blue) and downregulated (red) genes in the inulin diet group. **C** Significant enriched terms identified from KEGG Pathway analysis of the significantly up- and downregulated genes. **D** t-SNE plot with defined cell populations in colon epithelium summarizing data from single-cell RNA-Seq analysis of inulin diet fed mice and controls. **E** Heatmap of colon epithelial cell cluster markers colored by relative gene expression. Cell types are indicated by colored bars on top matching colors in (**D**). Select markers for each cluster are shown on the right of the heatmap. **F** Proportion of the absorptive, proliferative or secretory epithelial cell types in both dietary groups. T-test was used to calculate p-values and corrected by Benjamini- Hochberg false discovery rates. * *p* < 0.05. **G** Proportion of the 9 defined cell populations in both dietary groups. T-test was used to calculate p-values and corrected by Benjamini–Hochberg false discovery rates. * *p* < 0.05. **H** Frequency of cells expressing S or G2/M phase cell-cycle genes in total and in the main intestinal epithelial populations (EEC and Tuft cells were excluded because their numbers were very low). Results were analyzed using Fisher’s exact test. *p < 0.05, ***p < 0.001, ****p < 0.0001

To understand the effects of inulin in the colon epithelium in more detail, we performed bulk mRNA sequencing from extracted epithelial cells (IECs), which revealed 268 differentially expressed genes between the two experimental groups (Fig. 3A and B). Gene ontology analysis showed that cell cycle and DNA replication pathways were upregulated by the inulin diet, as well as genes involved in DNA repair including *Chek1*, *Exo1*, *Clspn*, and *Foxm1* (Figs. 3C and S3A). On the other hand, genes associated with the metabolism of lipids/fatty acids including *Acadl*, *Cpt1a* and *Hadha* were downregulated in the inulin diet group (Fig. 3C), a modulation that goes in line with the described effects of inulin consumption on lowering the circulating lipid profile and hepatic steatosis [38, 73]. The inulin diet also increased the expression of genes associated with differentiated epithelial cells, as shown by the upregulation of specific protein processing genes including *Uggt1*, *Pdia4*, *Ddost* and *Rpn1* (Fig. S3B). This result was linked with increased numbers of goblet cells in the inulin diet group, as indicated by higher expression of *Muc2* by IECs (Fig. S3C) and increased numbers of mucins-positive cells in the colon (Fig. S3D and E).

We next performed single cell RNAseq on colonic epithelial cells isolated from crypts of C57BL/6 mice kept on either control diet or inulin diet for 30 days to characterize the epithelial remodeling driven by the ingestion of inulin, and to better explore the transcriptional differences induced in the distinct cell types found in the epithelium. After removing low-quality and contaminant immune cells (described in the methods), we retained 15,432 EpCAM^+^ epithelial cells for further analysis. We identified 7 populations within the epithelial compartment: stem cells (4.6%), cycling transit-amplifying (TA) cells (6.5%), absorptive progenitors (7.2%), secretory epithelial cells (i.e., goblet [18,8%], enteroendocrine [0.8%] and tuft cells [0.7%]), and enterocytes (61.4%) (Fig. 3D). All populations were defined using well-characterized key markers (Figs. 3E and S3F; Table S2). Some subclusters could also be identified after an extra round of clustering, thereof enterocytes and goblet cells were further subdivided in immature (33.7%) and mature enterocytes (66.3% of total enterocytes) and immature (40.6%) and mature goblet cells (59.4%), respectively, based on distinct stages of expression of gene signatures described previously [60]. We compared the percentage of absorptive, proliferative, and secretory epithelial cells in both dietary groups and found that mice fed with inulin diet presented almost two times more proliferative cells compared to control, with no significant difference observed for absorptive or secretory cells (Fig. 3F). The proportion of intestinal epithelial cell populations was similar between both experimental groups (Fig. 3G).

Using a cell-cycle gene profile signature described previously [66, 74], we observed an increased proportion of cells in the G2/M phase in all cells combined (Total), as well as in stem cells, absorptive progenitors, enterocytes, and goblet cells from mice fed with inulin diet (Fig. 3H). Similarly, mice from the inulin diet group showed more stem cells and absorptive progenitors in S phase compared to the control animals (Fig. 3H). Our data also revealed that the inulin diet significantly increased the expression of genes associated with mucus production such as *Muc2*, *Fcgbp*, *Clca1*, and *Agr2* in mature and immature goblet cells (Fig. S3G). Goblet cells showed the highest number of differentially expressed genes (Fig. S3H). Together, these results indicate that the consumption of inulin impacts the proliferation and differentiation of colonic epithelial cells, increasing the number of cycling cells, the number of goblet cells and their expression of mucus-associated genes. This latter effect is in agreement with a recent paper that demonstrated increased mucus production in the colon of rats fed with inulin [49] and it will be explored by us in more detail in future studies.

### Epithelial proliferation induced by inulin diet is dependent on gut microbiota

**Fig. 4:**
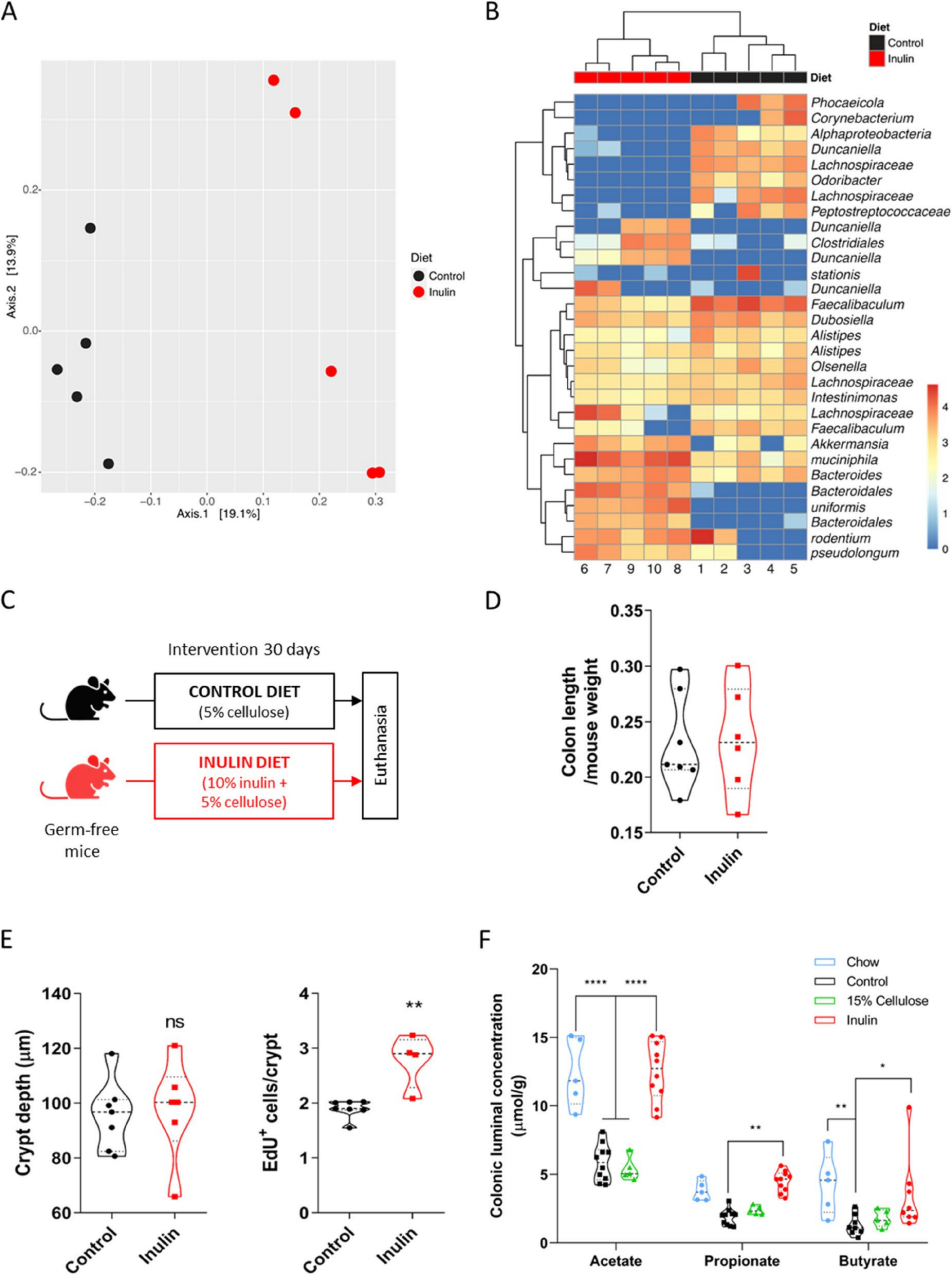
Epithelial proliferation induced by inulin diet is dependent on gut microbiota. **A** 16S rRNA gene sequencing of the C57BL/6 colon microbiota showing changes in beta diversity on inulin diet group. Expressed by UniFrac PCoA analysis (*n* = 6). PERMANOVA test (R^2^ = 0.1879, *p* = 0.012). **B** Heatmap of relative abundance taxa of bacteria in the different diets (*n* = 6), scale in log10. **C** Experimental model scheme with germ-free mice and two dietary groups. **D** Quantification of colon length normalized by mice weight (*n* = 6–7). Data pooled from 2 independent experiments. **E** Quantification of colon crypt depth (left) and number of EdU-positive cells per crypt (right) in germ-free mice (*n* = 4–7), the latter analyzed by Mann–Whitney test. Data pooled from 2 independent experiments. **F** Quantification of the levels of SCFAs in the colon fecal luminal content assessed by GC–MS (*n* = 5–10). Data pooled from 2 independent experiments. Results analyzed by two-way ANOVA. In all graphs, each point represents an individual animal. Unless otherwise stated, results were analyzed by Student’s t-test. *p < 0.05, **p < 0.01, ****p < 0.0001, and ns = not significant

As a soluble fiber and prebiotic, inulin is known to be fermented by the gut microbiota, mainly in the colon. The literature reports that inulin ingestion modulates the gut bacterial composition, with the promotion of a bifidogenic effect (increased abundance of *Bifidobacterium* spp.) being one of the most consistent alterations, although outcomes vary significantly among studies [34, 75]. In this regard, we aimed to characterize the bacterial community alterations caused by inulin ingestion in our experimental model. 16S rRNA gene analysis revealed that the inulin diet induced significant changes in the composition of the colon bacterial composition (Fig. 4A), with increased abundance of *Bacteroidetes* (*Bacteroides* spp., *Bacteroides uniformis*, and *Duncaniella* spp.), *Firmicutes* (*Clostridiales* spp.), *Verrucomicrobia* (*Akkermansia muciniphila*), and *Actinobacteria* (*Bifidobacterium pseudolongum*) species (Fig. 4B) relative to the control group. Linear discriminant Effect Size analysis (LEfSe) highlighted *Akkermansia* spp., *Duncaniella* spp., *Bacteroidales* spp., *Bacteroides* spp., and *Bifidobacterium* spp., as well as *Parasutterella* spp., *Clostridium XIVa*, *Eubacterium* spp., *Lawsonibacter* spp., *Rhodospirillales* spp., and *Butyricicoccus* spp. (Fig. S4A) with the greatest significance defining the differences between the two profiles of the gut microbiota.

To test whether the gut microbiota is required for inulin diet to induce the proliferative phenotype in the colon, germ-free (GF) mice were kept on control or inulin diet for 30 days (Fig. 4C). Under these conditions, ingestion of the inulin diet did not increase colon length and crypt depth, with animals fed on both diets showing similar colonic epithelial responses (Fig. 4D and E). Although there was a statistical difference in the number of EdU^+^ cells in GF mice fed with inulin diet compared to control (Fig. 4E), the impact was very limited (i.e. only one cell per crypt on average) and was not associated with changes in colon length or crypt depth. It is worth mentioning that EdU incorporation in the intestine of GF mice is very low compared to specific-pathogen free (SPF) mice and that we cannot exclude a possible effect of unfermented fibers in the colon of these animals, neither the participation of microbial components present on the diets used in this experiment (as shown by [76]) as our fiber sources were not tested for endotoxins or microbial contaminants. In addition, a control experiment using specific-pathogen free (SPF) Swiss mice (same background as the GF animals) confirmed that inulin was able to modulate intestinal proliferation in this mouse strain (Fig. S4B). In agreement, the depletion of the microbiota with antibiotics given in the drinking water also impaired the phenotypes induced by inulin consumption in C57BL/6 mice (Fig. S4C and D).

Short-chain fatty acids (SCFAs), important bacterial metabolites released by the gut microbiota after dietary fiber fermentation, are known for their strong impact on several components of the microbiome and the host [9]. In our model, measurement of acetate, propionate, and butyrate (the most abundant SCFAs produced in the colon) revealed a significant increase in their concentration in the colonic luminal content of mice fed with inulin diet relative to the control or 15% cellulose groups (Fig. 4F). The same increase was also observed in animals fed with chow diet (Fig. 4F), yet these mice did not present enhanced epithelial remodeling as those receiving inulin diet (Fig. S1J—L). We highlight that, although we could observe differences in the luminal concentration of these metabolites, no differences were seen when analyzing their molar ratio, which was approximately 3:1:1 (for acetate, propionate, and butyrate, respectively) for all dietary groups (Fig. S4I). Interestingly, inulin diet was also able to increase the colonic epithelial proliferation in mice lacking the FFAR2 receptor (FFAR2 KO), one of the main receptors for SCFAs in the intestine (Fig. S4E—H). Altogether, these experiments reveal the essential role of the gut microbiota in the colon epithelial remodeling driven by inulin and indicate that the axis SCFAs-FFAR2 may not be involved in this context.

### Fecal microbial transplantation recapitulates epithelial proliferation induced by the intake of inulin

**Fig. 5:**
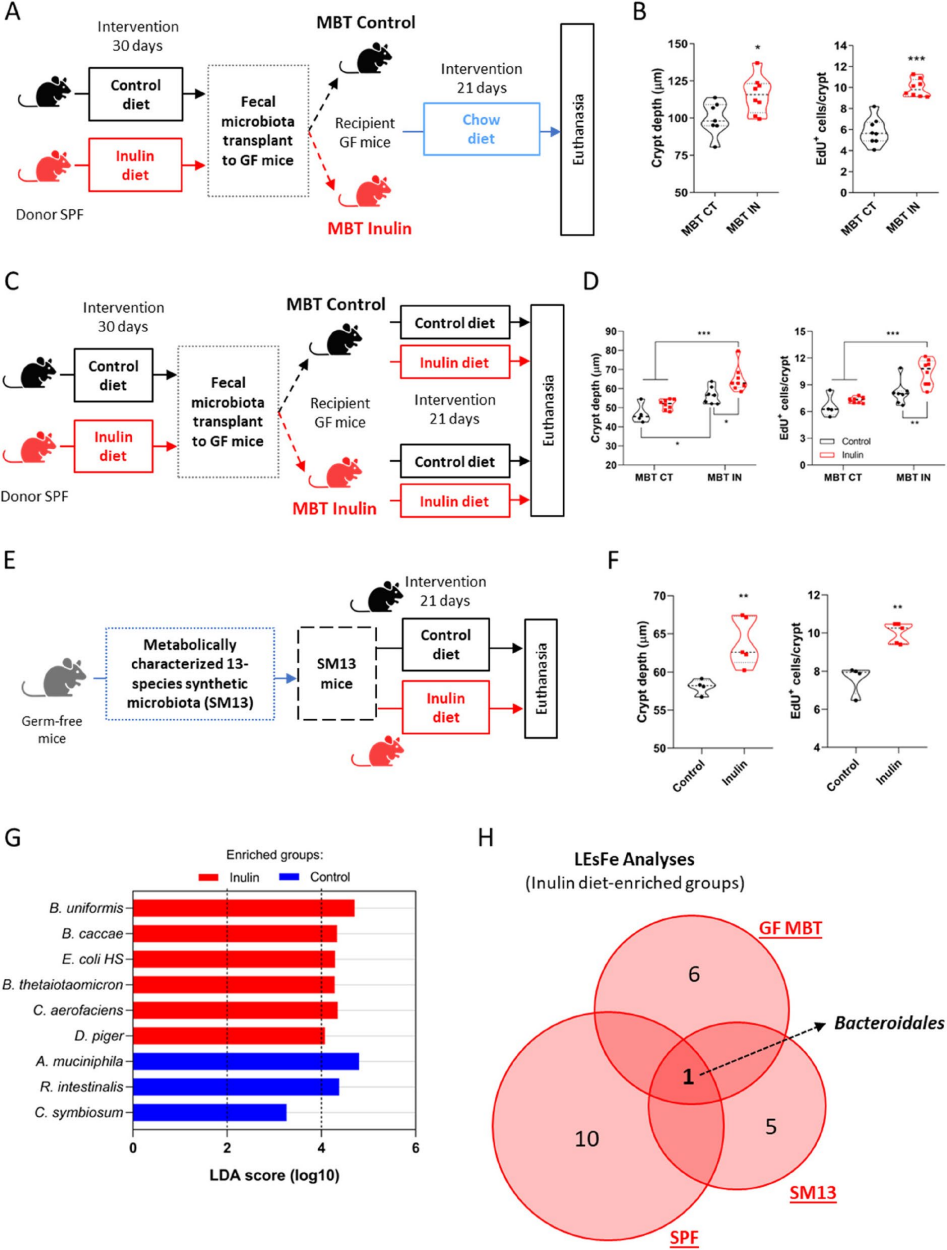
Fecal microbial transplantation recapitulates epithelial proliferation induced by the intake of inulin. **A** Schematic of the first fecal microbiota transplantation experiment (MBT), with SPF donors and GF recipient mice in the different diet conditions. **B** Quantification of colon crypt depth (left) and number of EdU-positive cells per crypt (right) (*n* = 7–8). **C** Schematic of the second fecal microbiota transplantation experiment (MBT). **D** Quantification of colon crypt depth (left) and number of EdU-positive cells per crypt (right) (*n* = 4–8). Results analyzed by two-way ANOVA. **E** Schematic of the gnotobiotic SM13 mice model (*n* = 4–5). **F** Quantification of colon crypt depth (left) and number of EdU-positive cells per crypt (right), the latter analyzed by Mann–Whitney test. **G** LEfSe analysis with LDA score of relative abundance taxa of bacteria of SM13 mice. **H** Venn diagrams showing the three distinct microbiota experiments and number of individual or shared bacterial groups obtained by LEfSe analysis of the inulin diet-enriched groups. SPF: specific pathogen-free mice, GF MBT: germ-free microbiota transplanted mice, SM13: gnotobiotic mice. In all graphs, each point represents an individual animal. Unless otherwise stated, results were analyzed by Student’s t-test. *p < 0.05, **p < 0.01, ***p < 0.001

To better understand the dynamics of the gut microbiota modulated by the inulin diet, we performed fecal microbiota transplants from SPF donors fed a control or inulin diet to recipient GF mice fed only with chow diet (Fig. 5A). We found that 21 days after colonization, the presence of the inulin diet-altered microbiota was able to induce and recapitulate the colonic epithelial proliferative profile compared to mice receiving microbiota from control-diet donors (Fig. 5B). 16S rRNA gene analysis revealed that the profile of the microbiota was still different between the two groups, even with the animals being kept on the same chow diet for three weeks after transplantation (Fig. S5A and B). LEfSe analysis revealed *Bacteroidales* showing changes with the strongest statistical significance related to inulin diet-transplanted mice (Fig. S5C), suggesting a potential role of this taxon in driving and/or maintaining the differences originally induced by inulin. Next, we repeated the fecal microbiota transplant experiment with both groups of recipient mice now being kept on either control or inulin diets after the transplantation (Fig. 5C). We observed that the received microbiota was a stronger factor driving the epithelial phenotype than the dietary condition that the animals received afterwards, as mice receiving control diet-altered microbiota presented similar proliferative rates regardless of the diet they were fed with, while mice receiving inulin diet-altered microbiota showed enhanced proliferation even when fed on the control diet (Figs. 5D and S5D).

To narrow down the complexity of the gut microbiome and better elucidate the relationship between inulin metabolization and the colonic epithelial remodeling, we used gnotobiotic SM13 mice colonized with a synthetic microbiota composed of only 13 fully sequenced species of commensal human gut bacteria, from all five of the gut-dominant phyla, with the capacity to degrade several distinct mono and polysaccharides (Table S3) [52]. After keeping these mice on control or inulin diet for 30 days (Fig. 5E), we found that the inulin diet still enhanced colonic epithelial proliferation even in the presence of such limited microbiota (Fig. 5F). 16S rRNA gene analysis showed modulations in 7 out of the 13 species, with three of these presenting enhanced relative abundance in the inulin group (*Bacteroides uniformis*, *Bacteroides caccae*, and *Collinsella aerofaciens*) (Fig. S5E). LEfSe analysis indicated *Bacteroides uniformis* as the most significantly altered species (Fig. 5G). In this sense, when comparing the LEfSe results of the bacterial groups enriched by the inulin diet, *Bacteroidales* was the only high abundance taxa occurring in all the three distinct experiments (i.e., SPF, GF fecal transplanted, and SM13 mice) (Fig. 5H). Taken together, these results highlight the direct role played by the altered microbiome in the induction and/or maintenance of colonic epithelial remodeling induced by the ingestion of inulin and suggest the involvement of specific members of this modulated bacterial community, such as possibly *Bacteroidales*.

### Cytokine IL-22 production is enhanced by inulin diet and is crucial for the epithelial proliferative phenotype

**Fig. 6:**
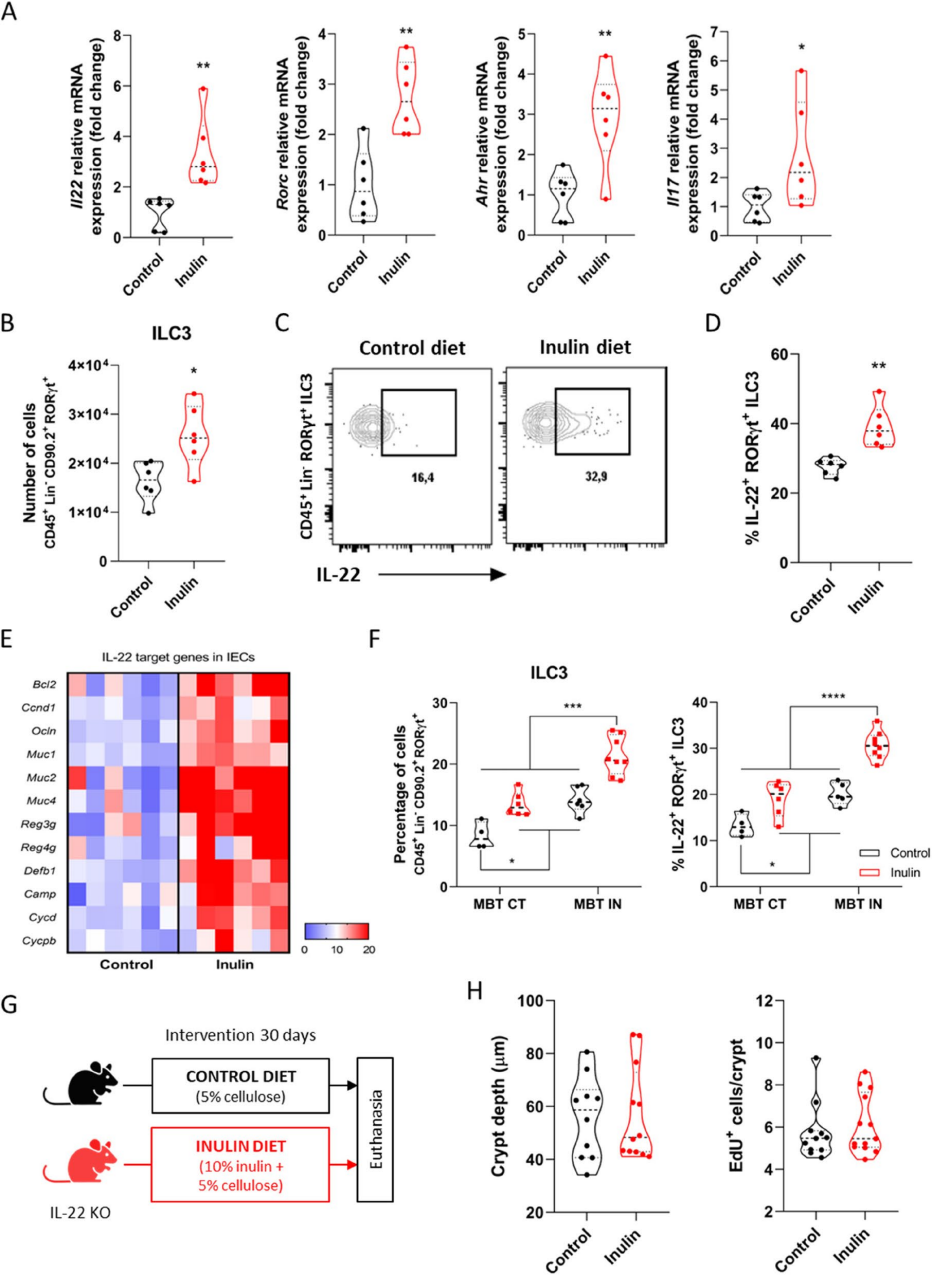
Cytokine IL-22 production is enhanced by inulin diet and is crucial for the epithelial proliferative phenotype. **A** Relative mRNA expression of *Il22*, *Rorc*, *Ahr* and *Il17* of colonic lamina propria lymphocytes by RT-qPCR (*n* = 6). **B**-**D**, **F** Flow cytometry analyses of colonic lamina propria immune cells (*n* = 4–6). **B** Quantification of ILC3s. **C** Gating strategy to define IL-22-positive events within the ILC3 population. **D** Quantification of the percentage of IL-22-positive ILC3s. **E** Heatmap with relative mRNA expression of IL-22-target genes of colonic epithelial cells by RT-qPCR (*n* = 6). **F** Quantification of the percentage of ILC3s (left) and IL-22-positive ILC3s (right). Results analyzed by two-way ANOVA. Related to fecal microbiota transplanted (MBT) mice described in Fig. 5C (*n* = 4–8). **G** Schematic of the experimental model with IL22 KO mice and the different diets (*n* = 10–12). **H** Quantification of colon crypt depth (left) and number of EdU-positive cells per crypt (right) of IL22 KO mice. Data were pooled from 2 independent experiments. Results analyzed by Mann–Whitney test. In all graphs, each point represents an individual animal. Unless otherwise stated, results were analyzed by Student’s t-test. *p < 0.05, **p < 0.01, ***p < 0.001, ****p < 0.0001

Interleukin-22 (IL-22) is a crucial cytokine that regulates gut homeostasis and host defense mechanisms, being produced in the intestine mostly by lamina propria (LP) T helper lymphocytes and innate lymphoid cells type 3 (ILC3s) [77–79]. The impact of IL-22 on epithelial proliferation has been described in different models [18, 80–86], although the precise cell target and mechanisms of action are still unclear [87]. In this sense, given this direct link, we next aimed to investigate whether IL-22 would be relevant in our model. Lymphocytes extracted from the colonic LP of mice fed with inulin diet showed increased expression of *Il22*, as well as genes related to IL-22, such as *Rorc, Ahr*, and *Il17* compared to those of control diet-fed animals (Fig. 6A). Flow cytometry analyses (Fig. S6A) showed that although the total number of T helper cells (Fig. S6B), ILC1s and ILC2s (Fig. S6C) did not change between the two diet groups, inulin diet increased the number of Th17 cells (Fig. S6D) and ILC3s (Fig. 6B). Moreover, both cell types also produced more IL-22 ex vivo compared to those from control mice (Figs. 6C, D and S6E). This effect was accompanied by an increase in the expression of IL-22 target genes in colonic IECs in the inulin group (Fig. 6E). A similar pattern was also observed in the colon LP of the microbiota-transplanted mice (Figs. 6F and S6F and G, related to mice from Fig. 5C).

Testing the hypothesis that IL-22 plays a pivotal role in the induction of a higher proliferative phenotype of the colon epithelium by inulin intake, we fed IL-22 knockout mice with either control or inulin diet for 30 days (Fig. 6G). Notably, in the absence of IL-22, inulin completely failed to induce any of the observed epithelial phenotypes (Figs. 6H and S6H—J). Together, these data show that inulin diet impacts the immune compartment in the colon lamina propria, increasing the production of IL-22 in this microenvironment, a requirement for the induction of colonic epithelial remodeling.

### γδ T cells, but not ILC3s or αβ T cells, are pivotal for induction of colonic epithelial remodeling by inulin ingestion

**Fig. 7:**
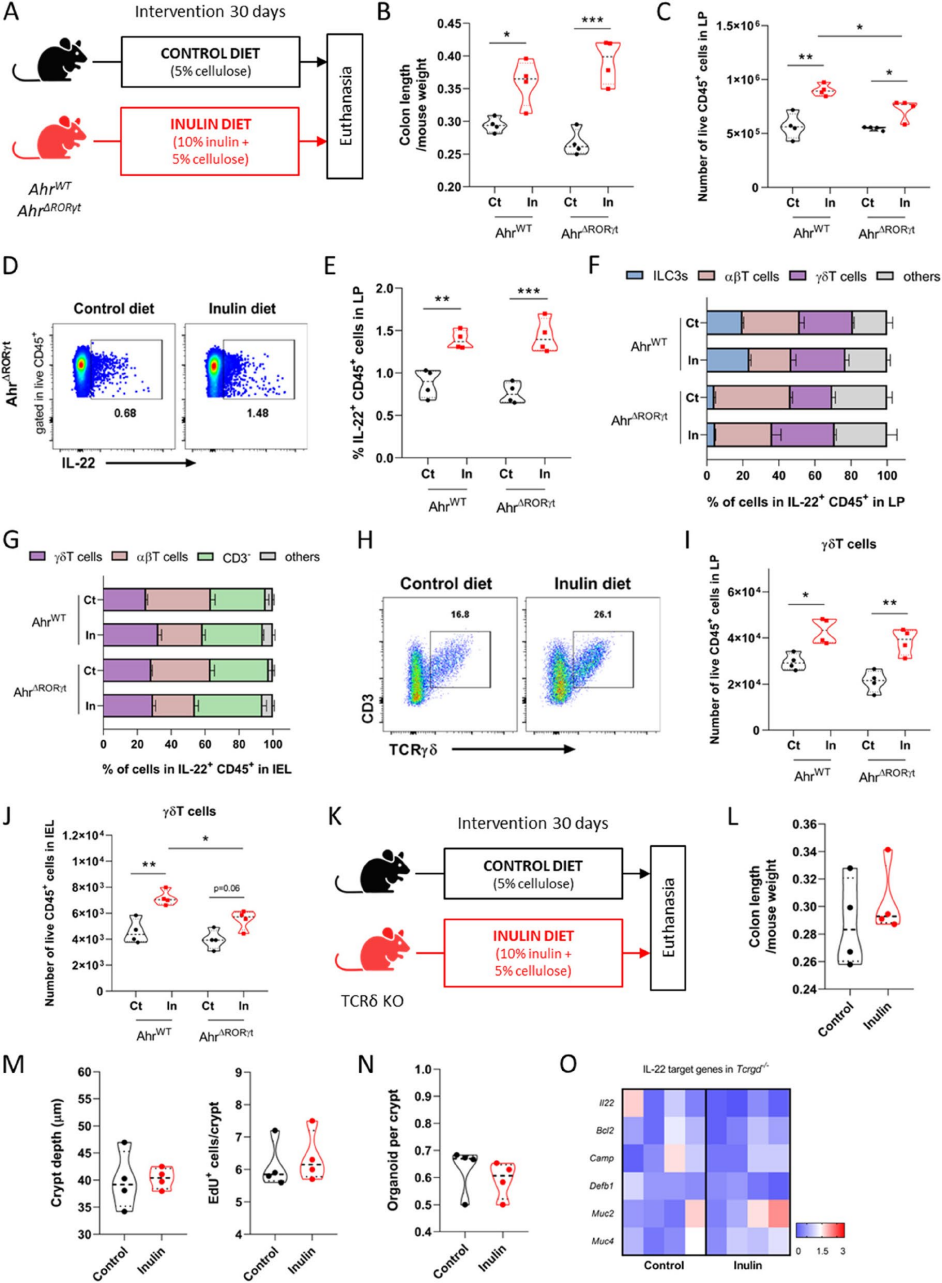
γδ T cells, but not ILC3s or αβ T cells, are pivotal for induction of colonic epithelial remodeling by inulin **A** Schematic of the experimental model with ILC3-deficient mice in the different diets (*n* = 4). **B** Quantification of the colon length of mice fed control (Ct) or inulin (In) diet. Results analyzed by two-way ANOVA. **C**-**J** Flow cytometry analyses of immune (CD45^+^) cells present in the colonic lamina propria (LP) or intraepithelial lymphocytes (IEL). **C** Quantification of CD45^+^ cells. Results analyzed by two-way ANOVA. **D** Gating strategy to define IL-22-positive events within the CD45^+^ population. **E** Quantification of the percentage of IL-22-positive CD45^+^ cells. Results analyzed by two-way ANOVA. **F** Percentage of IL-22 production by distinct cell types in the LP. Results analyzed by two-way ANOVA. **G** Percentage of IL-22 production by distinct cell types in the IEL. Results analyzed by two-way ANOVA. **H** Gating strategy to define the γδ T cell population. **I** Quantification of the γδ T cell population in the LP. Results analyzed by two-way ANOVA. **J** Quantification of the γδ T cell population within IEL. Results analyzed by two-way ANOVA. **K** Schematic of the experimental model with TCRδ KO mice in different diets (*n* = 4). **L** Quantification of colon length. **M** Quantification of colon crypt depth (left) and number of EdU-positive cells per crypt (right). **N** Quantification of clonogenicity capacity of colon crypts. Results analyzed by Mann–Whitney test. **O** Heatmap with relative mRNA expression of IL-22-target genes of colonic epithelial cells by RT-qPCR. In all graphs, each point represents an individual animal. Unless otherwise stated, results were analyzed by Student’s t-test. *p < 0.05, **p < 0.01, ***p < 0.001

Given the large contribution of ILC3s to the production of IL-22 in the intestine, we next used conditional knockout animals for the aryl hydrocarbon receptor (Ahr) in the RORγt compartment (hereby named *Ahr*^ΔRORγt^) to investigate the effects of inulin diet in this classic murine model of ILC3 deficiency [88]. Ahr regulates the survival and function of these cells [89]. In this sense, wild-type (*Ahr*^WT^) and ILC3-deficient (*Ahr*^ΔRORγt^) mice were fed control or inulin diet for 30 days (Fig. 7A) and colon immune cells from the LP and the intraepithelial lymphocyte (IEL) population were analyzed by flow cytometry. In agreement with the previous results, inulin increased ILC3s in the LP of *Ahr*^WT^ mice, while *Ahr*^ΔRORγt^ animals showed significantly lower numbers of ILC3s (shared in both diet groups) (Fig. S7A). Both in the presence or deficiency of ILC3s, inulin also led to longer colons (Fig. 7B) and showed similar effects on the number of CD45^+^ cells (Fig. 7C) in the LP, as well as on the number of IL-22 positive cells within CD45^+^ cells in the LP (Fig. 7D and E) and IEL (Fig. S7B). This data indicates that the deficiency of ILC3s did not significantly impact the phenotypes induced by the intake of inulin.

As IL-22 can also be produced by several distinct immunological cells in the gut including CD8^+^ T cells, γδ T cells, lymphoid tissue inducer cells, and potentially neutrophils [77, 90], we further examined production of this cytokine by different cell types. In the LP of *Ahr*^WT^ mice, most of the IL-22 came from T cells rather than ILC3s. Although the percentage of γδ T cells was not affected by inulin intake, these cells became the largest contributors to IL-22 production after the dietary intervention, as there was a significant reduction in the contribution of αβ T cells (Fig. 7F). This phenotype was even more pronounced in *Ahr*^ΔRORγt^ mice (Fig. 7F). The same parameters were analyzed in the IEL population. In *Ahr*^WT^ mice fed a control diet, most of the IL-22 came from αβ T cells, followed by CD3^−^ cells and γδ T cells. The inulin diet significantly reduced the contribution of αβ T cells and enhanced the contribution of γδ T cells, with no changes in the CD3^−^ and other populations (Fig. 7G). On the other hand, in *Ahr*^ΔRORγt^ mice, inulin diet did not affect the contribution from γδ T cells, but significantly reduced the contribution of αβ T cells, with the CD3^−^ cells becoming the predominant producers (Fig. 7G). We then tested the possibility that this T cell population would play a significant role in the epithelial remodeling induced by inulin intake in the colon. We found that ingestion of inulin diet increased the number of γδ T cells in both *Ahr*^WT^ and *Ahr*^ΔRORγt^ mice (Fig. 7H - J). The γδ T cells were the most significant producers of IL-22 ex vivo, and this was observed mainly in those cells coming from the IEL compartment of mice fed inulin diet (Fig. S7C).

Considering the relevance of the T lymphocytes on IL-22 production, we next tested the effects of inulin on *Rag1*^−/−^ mice (Fig. S7D), animals lacking most B and T cell populations (including the γδ T population) and with higher levels of ILC3s [91, 92]. In these *Rag1*^−/−^ mice, inulin diet did not induce any of the analyzed proliferative effects (Fig. S7E—H), indicating the importance of lymphocytes in this scenario and minimizing the role of ILC3s. Importantly, in TCRβ^−/−^ mice lacking only ɑβ T cells (Fig. S7I) inulin was still able to induce longer colons (Fig. S7J) and to increase the production of IL-22 by γδ T cell in the LP (Fig. S7K) and IEL (Fig. S7L). However, the same abrogation of any described epithelial responses induced by inulin intake could be reached by in vivo deletion of only the γδ T cell population, as demonstrated in TCRδ^−/−^ mice (Fig. 7K - O). Taken together, our data reveal that under homeostasis, inulin ingestion induces colonic epithelium remodeling through a mechanism involving IL-22 production and γδ T lymphocytes.

## Discussion

In this study, we elucidated the impact of a diet enriched with the soluble dietary fiber inulin on colonic epithelial barrier responses under the steady state and associated these effects to components of the gut microbiota and the local immune system. Consumption of inulin enhances the proliferative activity of intestinal stem cells, leading to increased cellular proliferative rate, deeper crypts, and longer colons. These effects occurred in an inulin dose-dependent manner and were also accompanied by an increase in goblet cell numbers and increased expression of mucus-associated genes. The gut microbiome was essential for the promotion of these epithelial responses induced by inulin intake, as we observed partial or absent phenotypes in germ-free or antibiotics-treated mice. We further demonstrated that this epithelial remodeling caused by inulin ingestion involves IL-22 and γδ T lymphocytes.

In many cases, the effects of fermentable fibers on gut physiology have been linked to the production and action of bacterial SCFAs [16, 93]. In our model, the elevated production of SCFAs was not always associated with the epithelial phenotype, as seen in the case of mice maintained on chow diet, and the SCFA-FFAR2 axis was not relevant to the epithelial response observed in mice after ingestion of inulin, suggesting that other mechanisms may be involved in the effects reported here. Fiber fermentation was necessary for the epithelial responses observed by us, as no response was seen after ingestion of a diet supplemented with a non-fermentable fiber (cellulose). The fermentation process leads to the generation of diverse bacterial-derived metabolites besides SCFAs, all playing important roles in distinct host cells and tissues [10]. A recent study performed metabolomic analyses of serum samples from control and inulin-fed mice and identified a vast number of modulated metabolites driven by inulin intake, in which bile acids, indoles and phenolic metabolites were among the most upregulated ones [44]. These metabolites may be directly or indirectly linked to the epithelial remodeling observed after ingestion of inulin. Secondary bile acids, for example, have been shown to support epithelial turnover at low concentrations, possibly via Wnt/β-catenin signaling [94–96], but to inhibit proliferation and to induce apoptosis at high levels, an effect that is dependent on FXR signaling [94, 97, 98]. Similarly, microbial indole derivatives can affect the activity of ISCs by different mechanisms [4], including modulation of AhR and β-catenin signaling [99], or indirectly through the induction of IL-22 in stromal lymphocytes [100].

Our microbiota analysis suggests a potential role of *Bacteroidales*, especially *Bacteroides* spp, in driving and/or maintaining the differences originally induced by inulin ingestion. Certain strains of *Bacteroides* possess the enzymes necessary to deconjugate bile salts into bile acids, as well as, converting primary bile acids produced by the liver into secondary bile acids through 7α-dehydroxylation [101, 102]. Secondary bile acids are more hydrophobic and detergent-like and have antimicrobial properties [103]. *Bacteroides* also conjugate bile acids by adding a sugar molecule, increasing their solubility, and making them less toxic to gut cells. Furthermore, *Bacteroides* are also able to produce indoles, through the breakdown of the amino acids such as tryptophan by tryptophanase [104]. These metabolites have been linked to the activation of AhR in gut-associated immune cells and subsequent production of IL-22 [105], a key cytokine for the epithelial remodeling observed in our study. In this sense, the production of indoles by gut bacteria may have an indirect beneficial effect on the host by regulating the immune response through the production of IL-22.

In fact, IL-22 is one of the most important molecules bridging the local immune system and the mucosal epithelium [87]. This cytokine has also been shown to protect ISCs from environmental genotoxic factors that can potentially disrupt genome integrity of cells, lead to epithelial barrier damage and ultimately to tumor formation [106]. Previous work led to the suggestion that the crypt structure itself protects stem cells from microbial-derived noxious molecules [107]. Based on our results, we hypothesize that the increased proliferation/differentiation and the generation of deeper crypts observed in mice fed with inulin could contribute to a mechanism protecting the colonic stem cells against toxic components generated by the normal, non-pathogenic microbiota.

Interestingly, IL-22 can have distinct and nonredundant roles in different models of intestinal infection and inflammation, probably due to specific niche localization [86]. A recent study demonstrated that ILCs are a more rapid source of IL-22 during acute states of injury, acting mostly in the surface epithelial cells. In contrast, T cells are the major source of IL-22 in chronic inflammation and/or late stages of infection, acting mostly in the epithelial cells located at the crypts and protecting these from invasion by the pathogenic bacterium *Citrobacter rodentium* [108]. Our results expand this notion, revealing that under steady state, the IL-22 produced by ILC3s and T cells plays a distinct role in the colon epithelial responses to commensal bacterial alterations. We demonstrated that although inulin intake increases the number of ILC3s and αβ T cells and their production of IL-22, this effect is not directly involved in the epithelial remodeling. Instead, we found that the γδ T lymphocytes-IL**-**22 axis was critical to regulate colonic stem cell function and, consequently, the epithelial modulations induced by inulin diet. This highlighted γδ T cells as a pivotal hub in the colonic diet-microbiota-epithelium crosstalk.

The role of γδ T lymphocytes in maintaining host homeostasis has been investigated by different groups, especially on mucosal surfaces [109–112]. However, the influence of both the gut microbiota and the diet on γδ T cells remains poorly understood [113]. While some studies have suggested that the intestinal microbiota and its metabolites can impact the number and functionality of γδ T lymphocytes [114, 115], others have shown that the expansion and activation of these cells occur via enterocytes signaling and are microbiota-independent [116, 117], with no specific bacteria being identified as inducers of γδ T cells in the intestinal lamina propria [117]. Nevertheless, the overall intestinal nutrient availability has been shown to alter the localization and response of γδ T cells [118], and the maintenance of γδ IELs seems to require some dietary compounds such AhR ligands [109]. Our results shed light in this context, demonstrating that ingestion of inulin alters the colon microenvironment, modulating the gut microbiota favoring commensals including *Bacteroidales*, which in turn affects the functionality of IL-22 producing γδ T lymphocytes, a pivotal local immune cell population that drives the adaptive and homeostatic remodeling of the colon epithelium in response to such luminal alterations.

Although our study provides new and exciting information about the adaptations of the intestinal epithelium to diet, several limitations should be considered. While our experiments with gnotobiotic mice and the 16S rRNA gene analysis indicate the involvement of specific microbiota members of the modulated bacterial community in the phenotype, additional experiments with germ free mono-colonized mice, metagenomic and metabolomic analysis are required to confirm the participation of specific components of the microbiota and to identify the signals involved in the phenotype. Second, even with increased number of goblet cells and changes at RNA level in genes relevant for mucus production, further analyses are necessary to characterize the effect on inulin's intake on the mucus layer including quantification of mucus production, an analysis that we were not able to perform for this study. Third, despite the fact that we observed a loss of phenotype in IL-22 and TCR delta knockout mice, the connection between these two aspects and the microbiota will need to be addressed in future studies. Fourth, we found that ingestion of other soluble fibers, such as pectin and fructooligosaccharide can also induce, at least to some extent, epithelial changes in mice similar to those observed with inulin. This was an unexpected finding, especially considering that pectin, for example, induces different modulations on the profile of the gut microbiota compared with inulin (data not shown). Future studies comparing the effects of different soluble fibers on the microbiota, epithelial and immune compartments will be relevant for addressing this aspect. Finally, the diet used in most of our experiments contained a high dose of inulin (10%). This concentration is similar or even lower than used in previous experimental studies [17, 18, 20, 39, 40] and we did not observe any harmful effect with it. However, the human dose equivalent, considering an intake of 3.5 g/day and the conversion formula described by [119], is around 1.4 g of inulin/kg (84 g of inulin/day for a person with 60 kgs). Such a dose is indeed too high when translated for human intake because of inulin’s side effects including bloating, flatulence, and intestinal discomfort with most of the clinical studies performed using doses of 10–30 g/day of this fiber [34]. Taking this into account, as well as the differences in gut microbial composition, dietary components and immunological responses between mouse and human [120–122], further studies will be needed to elucidate the role played by inulin intake in the remodeling of the intestinal epithelium in humans.

## Conclusion

Altogether, our study shows that the ingestion of inulin affects the proliferative activity of intestinal stem cells and drives a homeostatic remodeling of the colon epithelium (i.e., increased proliferation, deeper crypts, and increased production of mucus), an effect that requires the gut microbiota, γδ T cells, and the presence of IL-22. Understanding the physiological responses of the host to distinct dietary components and how the intestinal epithelium and local immune system communicate and adapt in a homeostatic manner to mutualistic members of the gut microbiota provides an opportunity to discover better and less invasive ways to clinically manipulate these intrinsic interactions. This also has potential to develop therapies supporting the maintenance of the steady state of a healthy individual, or treatments to ameliorate pathologies related to gastrointestinal disruptions.

## Supplementary Information

**Additional file 1: Table S1.** The composition of the two main diets used in this study. Related to Methods.

**Additional file 2: Table S2.** Cell markers for epithelial, immune and stromal cells diferentiation.

**Additional file 3:** **Table S3.** Gut bacterial community of the gnotobiotic SM13 mice.

**Additional file 4:** **Table S4.** Supplemental oligonucleotides. Related to Key Resources Table.

**Additional file 5: ** Supplemental Figures.

**Additional file 6: ** Raw and analyzed data generated during this study.

## Data Availability

All data generated or analyzed during this study are included in this published article (and its additional files). Requests for material should be made to the corresponding authors.mRNA sequencing data have been deposited at NCBI BioProject: PRJNA856648 Single-cell RNA sequencing data have been deposited at NCBI BioProject: PRJNA856646. 16S rRNA gene sequencing data have been deposited at NCBI BioProject: PRJNA862480.
