# Correction: Inulin diet uncovers complex diet-microbiota-immune cell interactions remodeling the gut epithelium

**DOI:** 10.1186/s40168-023-01579-x

**Published:** 2023-05-31

**Authors:** Renan Oliveira Corrêa, Pollyana Ribeiro Castro, José Luís Fachi, Vinícius Dias Nirello, Salma El‑Sahhar, Shinya Imada, Gabriel Vasconcelos Pereira, Laís Passariello Pral, Nathália Vitoria Pereira Araújo, Mariane Font Fernandes, Valquíria Aparecida Matheus, Jaqueline de Souza Felipe, Arilson Bernardo dos Santos Pereira Gomes, Sarah de Oliveira, Vinícius de Rezende Rodovalho, Samantha Roberta Machado de Oliveira, Helder Carvalho de Assis, Sergio Costa Oliveira, Flaviano Dos Santos Martins, Eric Martens, Marco Colonna, Patrick Varga‑Weisz, Marco Aurélio Ramirez Vinolo

**Affiliations:** 1 Laboratory of Immunoinflammation, Department of Genetics, Evolution, Microbiology, and Immunology, Institute of Biology, University of Campinas, Campinas, SP 13083-862 Brazil; 2 Koch Institute for Integrative Cancer Research at MIT, Cambridge, MA 02139 USA; 3 Department of Pathology and Immunology, Washington University School of Medicine, Saint Louis, MO 63110 USA; 4 International Laboratory for Microbiome Host Epigenetics, Department of Genetics, Evolution, Microbiology, and Immunology, Institute of Biology, University of Campinas, Campinas, SP 13083-862 Brazil; 5 School of Life Sciences, University of Essex, Colchester, CO4 3SQ UK; 6 Department of Gastroenterological and Transplant Surgery, Graduate School of Biomedical and Health Sciences, Hiroshima University, Hiroshima, 734-8551 Japan; 7 University of Michigan Medical School, Ann Arbor, MI 48109 USA; 8 Laboratory of Biotherapeutics Agents, Department of Microbiology, Institute of Biological Sciences, Federal University of Minas Gerais, Belo Horizonte, MG 31270-901 Brazil; 9 Department of Biochemistry and Immunology, Institute of Biological Sciences, Federal University of Minas Gerais, Belo Horizonte, MG 31270-901 Brazil; 10 Department of Immunology, Institute of Biomedical Sciences, University of São Paulo, São Paulo, SP 05508-000 Brazil; 11 São Paulo Excellence Chair, Department of Genetics, Evolution, Microbiology, and Immunology, Institute of Biology, University of Campinas, Campinas, SP 13083-862 Brazil; 12 Experimental Medicine Research Cluster, Campinas, SP 13083-862 Brazil; 13 Obesity and Comorbidities Research Center (OCRC), University of Campinas, Campinas, SP 13083-864 Brazil

**Correction: Microbiome 11, 90 (2023)**

**https://doi.org/10.1186/s40168-023-01520-2**

Following publication of the original article [1], the author reported that the caption for additional file figures were incorrect and that an additional file is missing. The caption captured for the additional file figures should be the caption of the missing file, that is, the Raw and analyzed data.

The original article has been updated.
