# Supplementary material for: Inulin diet uncovers complex diet-microbiota-immune cell interactions remodeling the gut epithelium
Source: Microbiome. 2023 Apr 26;11:90. doi: 10.1186/s40168-023-01520-2 (PMC10131329; doi:10.1186/s40168-023-01520-2)
Supplement: Supplementary file 2 — Additional file 1: Table S1. The composition of the two main diets used in this study. Related to Methods. [file 40168_2023_1520_MOESM1_ESM.docx]

| Table S1. The composition of the two main diets used in this study. Related to Methods | | | | |
| --- | --- | --- | --- | --- |
|  | **Control Diet**  **(D10012-M)** | | **Inulin Diet**  **(D19071901)** | |
|  | **gm%** | **kcal%** | **gm%** | **kcal%** |
| Protein | 14 | 15 | 13 | 15 |
| Carbohydrate | 73 | 76 | 75 | 76 |
| Fat | 4 | 9 | 4 | 9 |
| Total |  | 100 |  | 100 |
| kcal/gm | **3.85** |  | **3.61** |  |
|  | | | | |
| Ingredients | **gm** | **kcal** | **gm** | **kcal** |
| Casein | 140 | 560 | 140 | 560 |
| L-Cystine | 1.8 | 7 | 1.8 | 7 |
|  |  |  |  |  |
| Corn Starch* | 495.692 | 1983 | 455.6 | 1822 |
| Maltodextrin 10 | 125 | 500 | 125 | 500 |
| Sucrose | 100 | 400 | 100 | 400 |
|  |  |  |  |  |
| Cellulose, BW200** | 50 | 0 | 50 | 0 |
| Inulin, Orafti HP*** | 0 | 0 | 106.95 | 160 |
|  |  |  |  |  |
| Soybean Oil | 40 | 360 | 40 | 360 |
|  |  |  |  |  |
| t-Butylhydroquinone | 0.008 | 0 | 0.008 | 0 |
|  |  |  |  |  |
| Mineral Mix S10022M | 35 | 0 | 35 | 0 |
|  |  |  |  |  |
| Vitamin Mix V10037 | 10 | 40 | 10 | 40 |
| Choline Bitartrate | 2.5 | 0 | 2.5 | 0 |
|  | | | | |
| Total | **1000** | **3850** | **1066.86** | **3850** |

1. The diets containing 15% Cellulose, 2% Inulin, 5% Inulin, 20% Inulin, 10% Pectin and 10% FOS were prepared following the same formulation above with the necessary adaptations in the values of corn starch (*), cellulose (**) and inulin (***) to match their specific fiber concentrations.
2. Conventional diet is the standard animal facility chow (Nuvilab CR-1).
