# Supplementary material for: Inulin diet uncovers complex diet-microbiota-immune cell interactions remodeling the gut epithelium
Source: Microbiome. 2023 Apr 26;11:90. doi: 10.1186/s40168-023-01520-2 (PMC10131329; doi:10.1186/s40168-023-01520-2)
Supplement: Supplementary file 4 — Additional file 3: Table S3. Gut bacterial community of the gnotobiotic SM13 mice. [file 40168_2023_1520_MOESM3_ESM.docx]

| **Table S3. Gut bacterial community of the gnotobiotic SM13 mice** | | |
| --- | --- | --- |
| **PHYLUM** | **SPECIES** | **ABILITY TO GROW *IN VITRO* IN THE PRESENCE OF INULIN AS THE ONLY CARBON SOURCE** |
| *Bacteroidetes* | *Bacteroides ovatus* | **YES** |
|  | *Bacteroides uniformis* | **YES** |
|  | *Bacteroides thetaiotaomicron* | **YES** |
|  | *Bacteroides caccae* | **YES** |
|  | *Barnesiella intestinihominis* | NO |
| *Firmicutes** | *Roseburia intestinalis* | **YES** |
|  | *Eubacterium rectale* | **YES** |
|  | *Marvinbryantia formatexigens* | **YES** |
|  | *Clostridium symbiosum* | NO |
| *Actinobacteria* | *Collinsella aerofaciens* | NO |
| *Proteobacteria* | *Escherichia coli* HS | NO |
|  | *Desulfovibrio piger* | NO |
| *Verrucomicrobia* | *Akkermansia muciniphila* | NO |

* We highlight the fact that the original community design by Desai et al. (2016) contained 14 species of bacteria, including *Faecalibacterium prausnitzii*. However, our 16S analysis revealed its absence, indicating that such fastidious *Firmicutes* was not able to colonize our mice. This could have happened directly in the parental generation, or this bacterium was only not able to be transmitted to the pups.
