# Supplementary material for: Inulin diet uncovers complex diet-microbiota-immune cell interactions remodeling the gut epithelium
Source: Microbiome. 2023 Apr 26;11:90. doi: 10.1186/s40168-023-01520-2 (PMC10131329; doi:10.1186/s40168-023-01520-2)
Supplement: Supplementary file 5 — Additional file 4: Table S4. Supplemental oligonucleotides. Related to Key Resources Table. [file 40168_2023_1520_MOESM4_ESM.docx]

| **Table S4. Supplemental oligonucleotides. Related to Key Resources Table** | | |
| --- | --- | --- |
| **OLIGONUCLEOTIDES** | **SOURCE** | **IDENTIFIER** |
| ***Ahr*:** 5’GCCCTTCCCGCAAGATGTTAT-3’ and 5’GCTGACGCTGAGCCTAAGAAC-3’ |  | N/A |
| ***Bcl2*:** 5’TGA​GTA​CCT​GAA​CCG​GCA​TCT-3’ and 5’GCA​TCC​CAG​CCT​CCG​TTA​T-3’ |  | N/A |
| ***Camp*:** 5’TCTCTACCGTCTCCTGGACCTG-3’ and 5’CCACATACAGTCTCCTTCACT-3’ |  | N/A |
| ***Ccnd1*:** 5’GCA​AGC​ATG​CAC​AGA​CCT​T-3’ and 5’GTT​GTG​CGG​TAG​CAG​GAG​A-3’ |  | N/A |
| ***Cycd*:** 5’GCAAGCATGCACAGACCTT-3’ and 5’GTTGTGCGGTAGCAGGAGA-3’ |  | N/A |
| ***Cycpb*:** 5’CAAGCTGAAGCACTACG-3’ and 5’AGGCCGTTCTAGCTTC-3’ |  | N/A |
| ***Defb1*:** 5’CCAGATGGAGCCAGGTGTTG-3’ and 5’CTGGAGCGGAGACAGAATCC-3’ |  | N/A |
| ***Il17:*** 5’TCAGCGTGTCCAAACACTGAG-3’ and 5’GACTTTGAGGTTGACCTTCACAT-3’ |  | N/A |
| ***Il22:*** 5’AGA​ATG​TCA​GAA​GGC​TGA​AGG​CG-3’ and 5’AGG​AGC​AGT​TCT​TCG​TTT​TCT​AG-3’ |  | N/A |
| ***Muc1*:** 5’CCC​TAC​CTA​CCA​CAC​TCA​CGG​ACG-3’ and 5’GTG​GTC​ACC​ACA​GCT​GGG​TTG​GT-3’ |  | N/A |
| ***Muc2*:** 5’CGACTGTGAGCAGTGTGTCA-3’ and 5’GGGTAGGGTCACCTCCATCT-3’ |  | N/A |
| ***Muc4*:** 5’GAG​GGC​TAC​TGT​CAC​AAT​GGA​GGC-3’ and 5’AGG​GTT​CCG​AAG​AGG​ATC​CCG​TAG-3’ |  | N/A |
| ***Reg3g*:** 5’TTC​CTG​TCC​TCC​ATG​ATC​AAA​A-3’ and 5’CAT​CCA​CCT​CTG​TTG​GGT​TCA-3’ |  | N/A |
| ***Reg4g*:** 5’CTGGAATCCCAGGACAAAGAGTG-3’ and 5’CTGGAGGCCTCCTCAATGTTTGC-3’ |  | N/A |
| **ROR-γt (*Rorc)*:** 5’TCC​ACT​ACG​GGG​TTA​TCA​CCT-3’ and 5’AGT​AGG​CCA​CAT​TAC​ACT​GCT-3’ |  | N/A |
| **β*2m*:** 5’CCC​CAC​TGA​GAC​TGA​TAC​ATA​CG-3’ and 5’CGA​TCC​CAG​TAG​ACG​GTC​TTG-3’ |  |  |
| **16S Eubacteria:** 5’ACT​CCT​ACG​GGA​GGC​AGC​AGT -3’ and 5’ATTACCGCGGCTGCTGGC-3’ |  | N/A |
| **16S rRNA Sequence Primers:** 5’TCGTCGGCAGCGTCAGATGTGTATAAGAGACA  GCCTACGGGNGGCWGCG-3’ and 5’ GTCTCGTGGGCTCGGAGATGTGTATAAGAGAC  AGGACTACHVGGGTATCTAATCC-3’ |  | N/A |
