# Supplementary material for: Inulin diet uncovers complex diet-microbiota-immune cell interactions remodeling the gut epithelium
Source: Microbiome. 2023 Apr 26;11:90. doi: 10.1186/s40168-023-01520-2 (PMC10131329; doi:10.1186/s40168-023-01520-2)
Supplement: Supplementary file 6 — Additional file 5: Supplemental Figures. [file 40168_2023_1520_MOESM5_ESM.docx]

**SUPPLEMENTAL FIGURES**


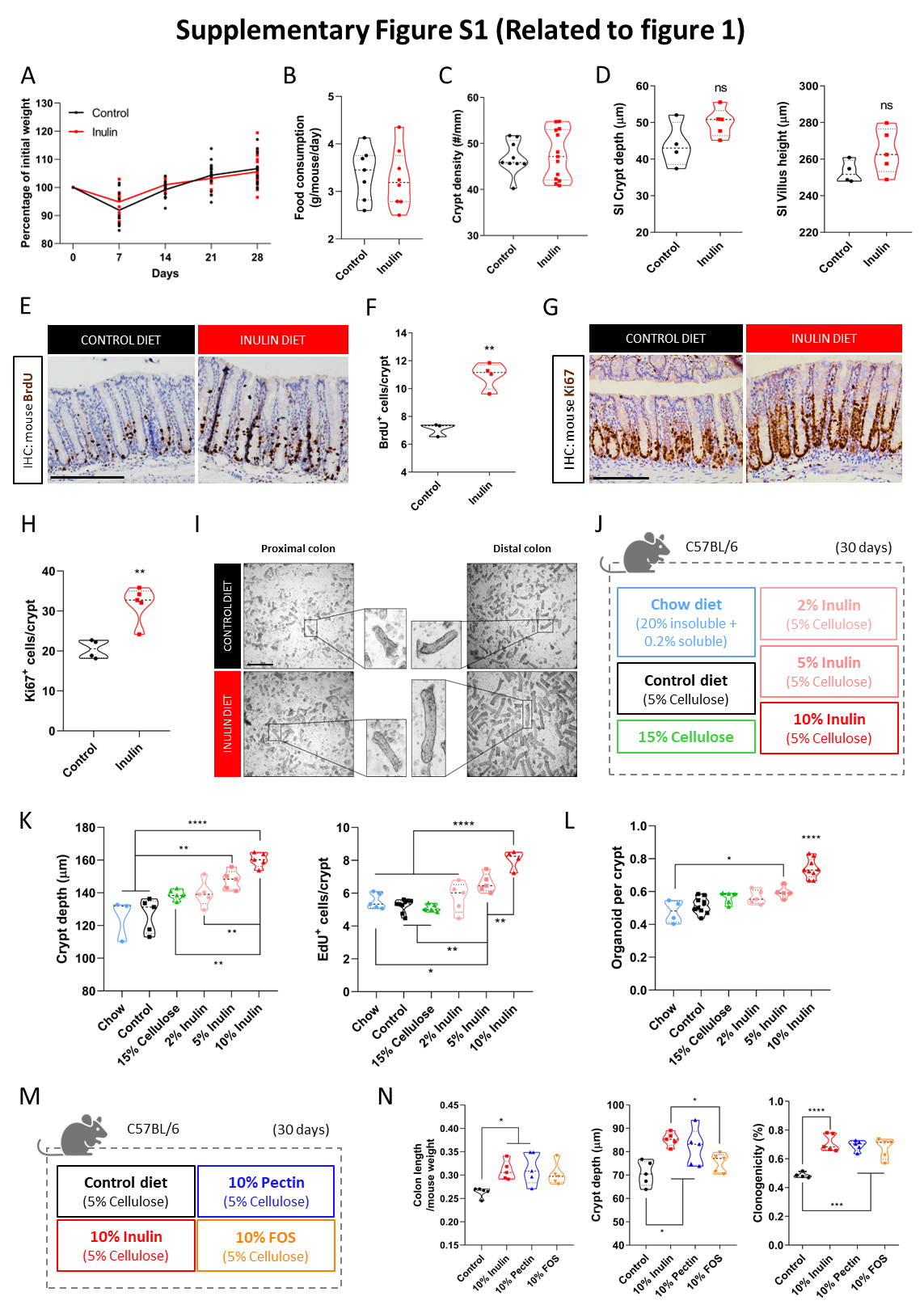


**Supplementary Figure 1 (Related to Figure 1)**

(A) Body mass variation over time of mice fed with control or diet (n = 12-19). Data were pooled from 3 independent experiments.

(B) Measurement of food intake, per mouse, over time (n = 7-8). Data were pooled from 2 independent experiments.

(C) Measurement of colon crypt density (n = 9-13). Data were pooled from 2 independent experiments.

(D) Measurement of small intestine crypt depth (left) and villus height (right) (n = 4-5). Results analyzed by Mann-Whitney test.

(E) Visualization of BrdU-positive cells in colonic crypts by optical microscopy following staining with anti-BrdU antibody. Scale bars, 100 µm.

(F) Quantification of BrdU-positive cells per crypt (n = 3-4).

(G) Visualization of Ki67-positive cells in colonic crypts by optical microscopy following staining with anti-Ki67 antibody. Scale bars, 50 µm.

(H) Quantification of Ki67-positive cells per crypt (n = 4-5).

(I) Representative optical microscope images of isolated crypts from proximal and distal colon. Scale bars, 200 µm.

(J) Experimental model scheme with six distinct dietary groups.

(K) Quantification of colon crypt depth (left) and number of EdU-positive cells per crypt (right) (n = 3-9). Results were analyzed by one-way ANOVA.

(L) Quantification of clonogenicity capacity of colon crypts (n = 4-9). Results were analyzed by one-way ANOVA.

(M) Experimental model scheme of mice fed with different diets enriched with distinct soluble fibers.

(N) Quantification of normalized colon length (left), colon crypt depth (middle) and clonogenicity capacity of colon crypts (left) (n = 4-5). Results were analyzed by one-way ANOVA.

In all graphs, each point represents an individual animal. Unless otherwise stated, results were analyzed by Student’s t-test. *p < 0.05, **p < 0.01, ***p < 0.001, ****p < 0.0001, and ns = not significant.


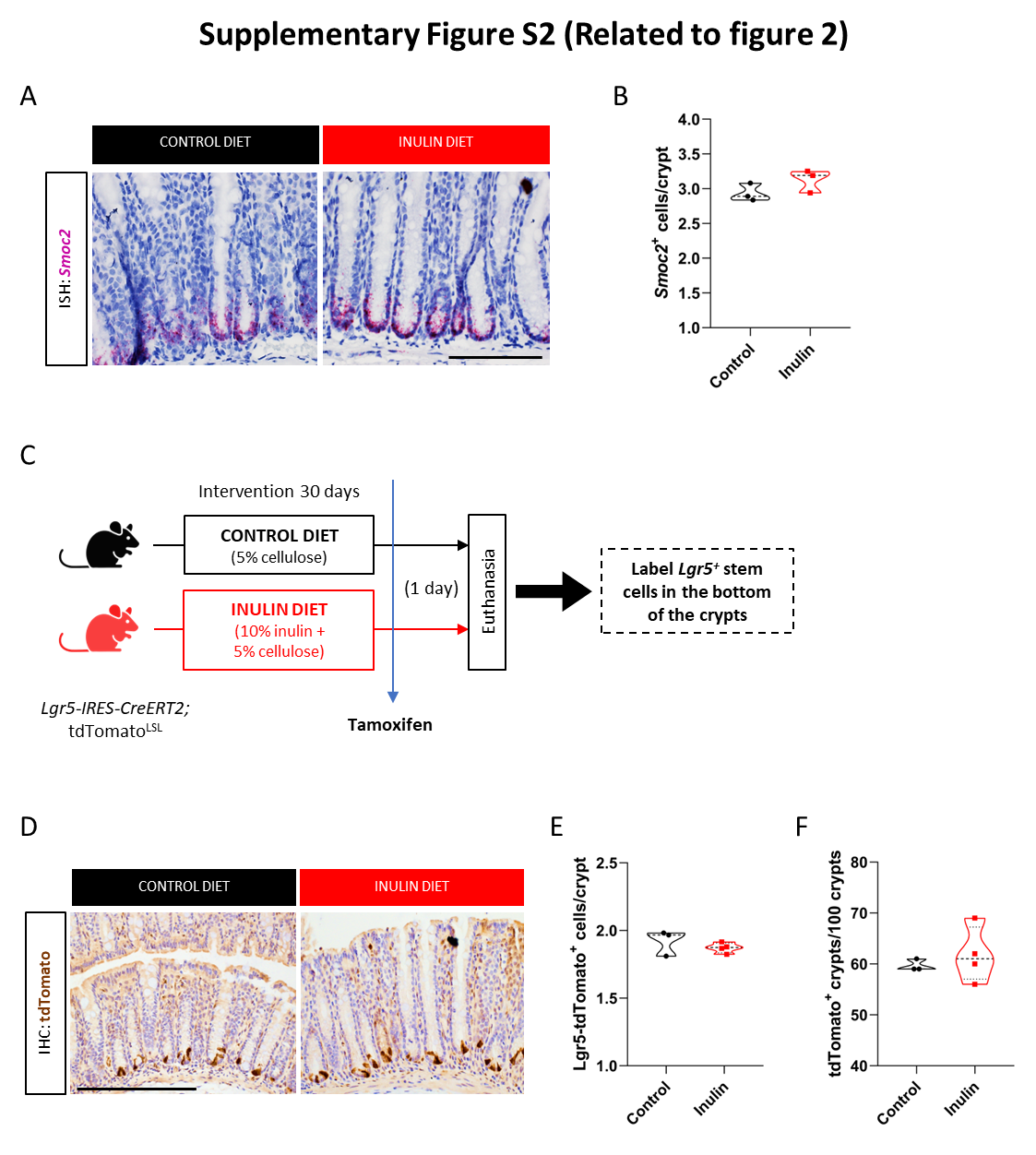


**Supplementary Figure 2 (Related to Figure 2)**

(A) Visualization of *Smoc2* *in situ* hybridization in colonic crypts by optical microscopy. Scale bars, 50 µm.

(B) Quantification of the number of *Smoc2*-positive cells per crypt (n = 3).

(C) Experimental model scheme with lineage-tracer mice and short-term (1 day) tamoxifen injection (n = 3-4).

(D) Visualization of tdTomato-positive cells in the base of colonic crypts by optical microscopy following staining with anti-tdTomato antibody. Scale bars, 100 µm.

(E) Quantification of tdTomato-positive cells per crypt.

(F) Quantification of tdTomato-positive crypts.

In all graphs, each point represents an individual animal. Results were analyzed by Student’s t-test.

**
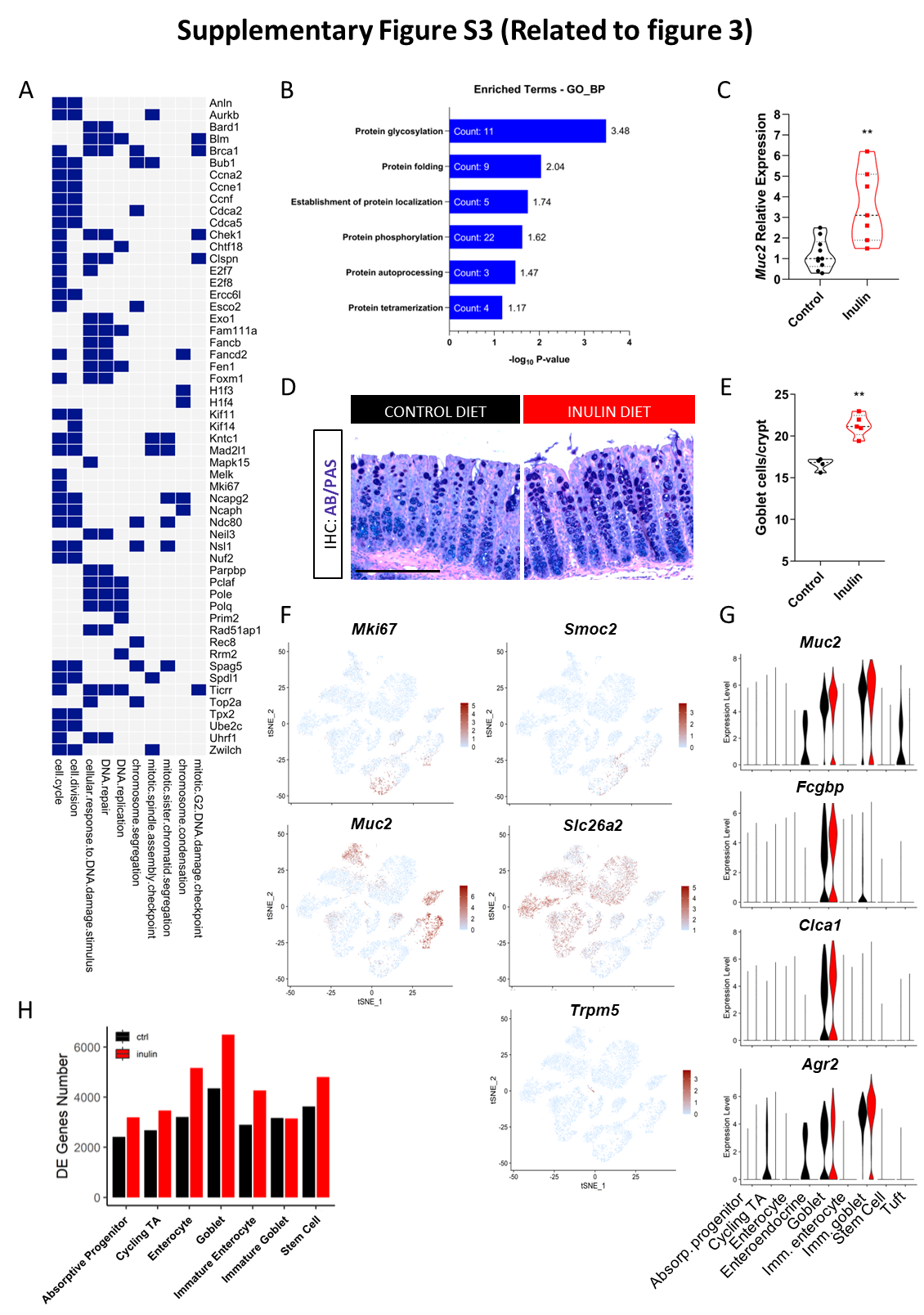
**

**Supplementary Figure 3 (Related to Figure 3)**

(A) Graph displaying significantly upregulated genes related to DNA repair in colon epithelial cells after transcriptome analysis.

(B) Significant enriched terms identified from gene ontology (GO) analysis of significantly upregulated genes in the inulin group after transcriptome analysis.

(A and B) DESeq statistical test with Benjamin-Hochberg correction. Significance when p <0.05.

(C) *Muc2* expression in colon epithelial cells determined by RT-qPCR (n = 7-10). Data were pooled from 2 independent experiments.

(D) Representative optical microscope images of colonic epithelium stained with Alcian blue/Periodic acid-Schiff. Scale bars, 100 μm.

(E) Quantification of goblet cells per colonic crypt (n = 4-5).

(F) tSNE map showing the combination of all cells expressing some of the main population markers.

(G) Expression of mucus-associated genes in distinct epithelial cell populations as determined by scRNA-seq.

(H) Graph displaying number of differentially expressed (DE) genes (up- and downregulated) by each epithelial cell subpopulation after single cell transcriptome analysis. MAST statistical test, p-value cutoff 0.01.

In all graphs, each point represents an individual animal. Results were analyzed by Student’s t-test. **p < 0.01.

**
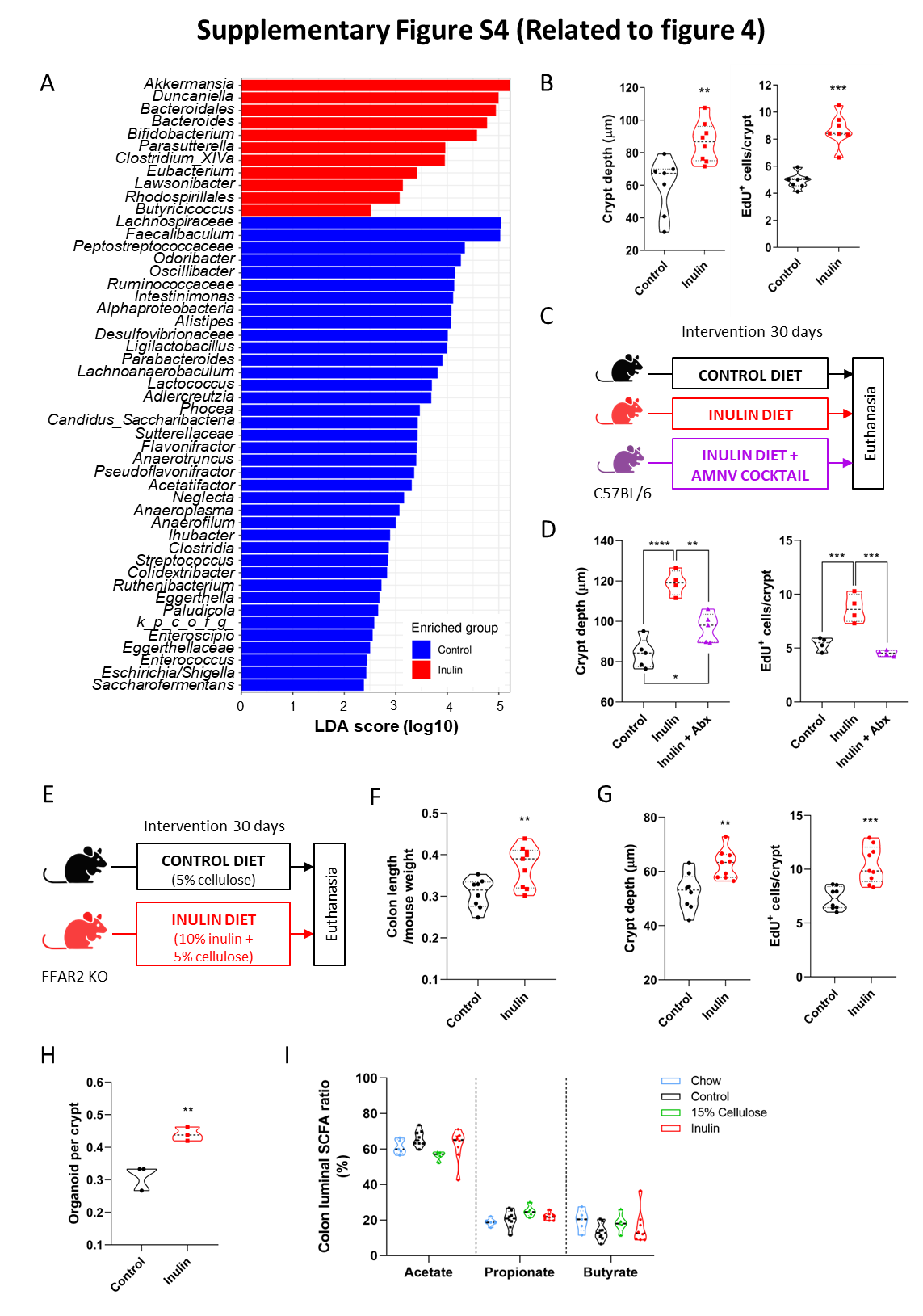
**

**Supplementary Figure 4 (Related to Figure 4)**

(A) LEfSe analysis with LDA score of relative abundance of taxa of bacteria measured in colon luminal fecal content of C57BL/6 mice fed control or inulin diet using 16S rRNA gene analysis.

(B) Quantification of colon crypt depth (left) and number of EdU-positive cells per crypt (right) of Swiss SPF mice (n = 7-8).

(C) Schematic of antibiotic treatment given in the drinking water in C57BL/6 mice together with two different diets (n = 4-5).

(D) Quantification of colon crypt depth (left) and number of EdU-positive cells per crypt (right). Results analyzed by one-way ANOVA.

(E) Experimental model scheme with FFAR2 KO mice (n = 8-9).

(F) Quantification of colon length.

(G) Quantification of colon crypt depth (left) and number of EdU-positive cells per crypt (right).

(H) Quantification of clonogenicity capacity of colon crypts (n = 3).

(I) SCFAs molar ratio in colon fecal luminal content (n = 5-10). Data pooled from 2 independent experiments. Results analyzed by two-way ANOVA.

In all graphs, each point represents an individual animal. Unless otherwise stated, results were analyzed by Student’s t-test. *p < 0.05, **p < 0.01, ***p < 0.001, and ****p < 0.0001.

**
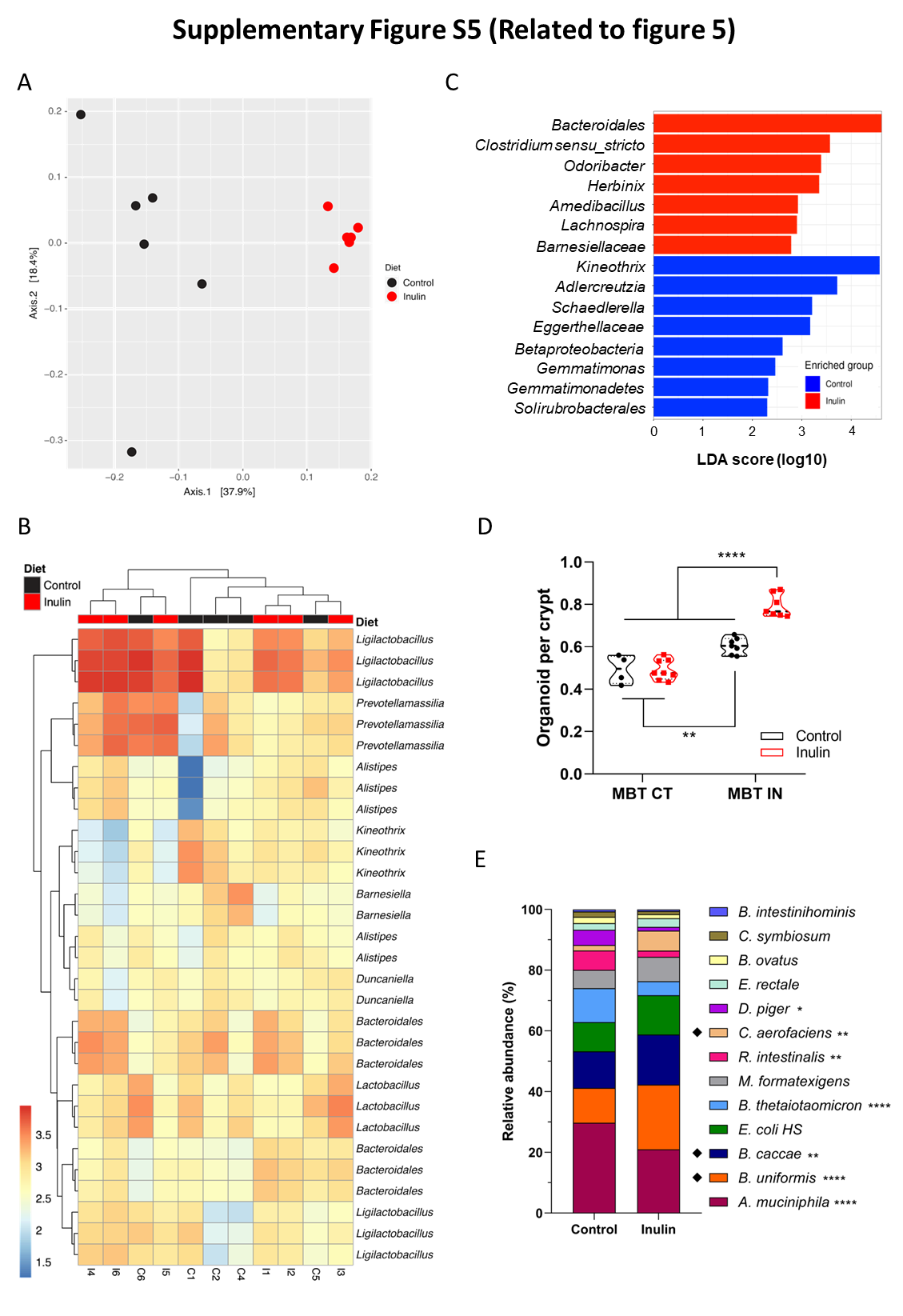
**

**Supplementary Figure 5 (Related to Figure 5)**

(A-C) Microbiota composition of colon luminal fecal content from microbiota transplanted (MBT) mice was analyzed by 16S rRNA gene sequencing (n = 5-6). Related to mice described in Fig. 5 A.

(A) Beta diversity of gut microbiota expressed by UniFrac PCoA analysis. PERMANOVA test (R^2^ = 0.3599, p = 0.002).

(B) Heatmap with relative abundance taxa of bacteria, log10 scale.

(C) LEfSe analysis with LDA score of relative abundance taxa of bacteria measured in colon luminal fecal content of mice.

(D) Quantification of clonogenicity capacity of colon crypts (n = 3-4). Results analyzed by two-way ANOVA. Related to fecal microbiota transplanted mice described in Fig. 5 C.

(E) Relative abundance taxa of bacteria measured in colonic luminal content of SM13 mice after the two different diets. * indicates statistical difference between both dietary groups; ✦ indicates species with enhanced abundance in the inulin group.

In all graphs, each point represents an individual animal. *p < 0.05, **p < 0.01, and ****p < 0.0001.

**
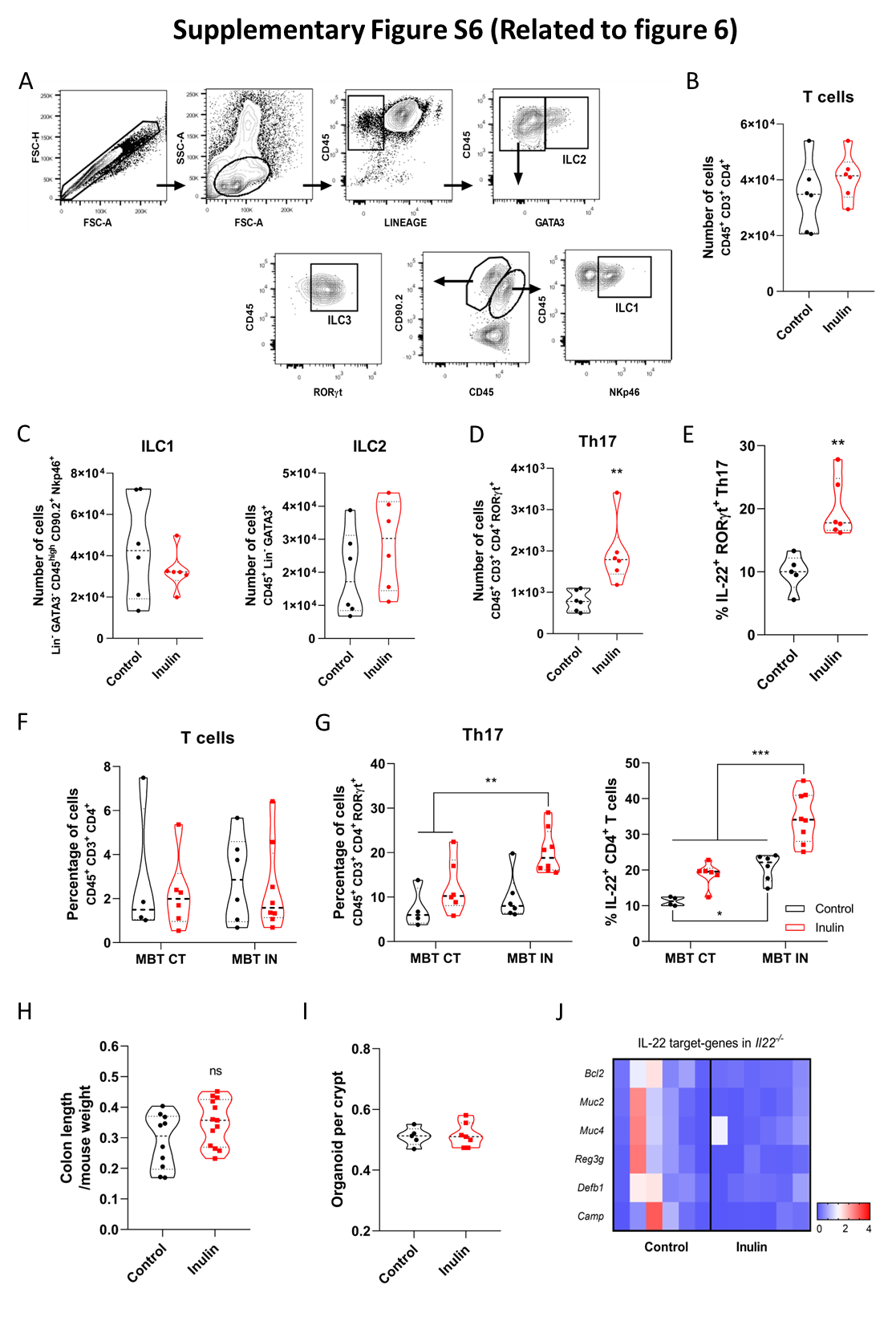
**

**Supplementary Figure 6 (Related to Figure 6)**

(A) Gate strategies to define the ILC populations in colon lamina propria (n = 6).

(B) Quantification of T cells.

(C) Quantification of ILC1s (right) and ILC2s (left).

(D) Quantification of Th17 cells.

(E) Quantification of IL-22-positive Th17 cells.

(F-G) Analysis of colonic lamina propria. Related to fecal microbiota transplanted mice described in Fig. 5C.  Results analyzed by two-way ANOVA (n = 4-8).

(F) Quantification of T cells.

(G) Quantification of Th17 cells (left) and IL-22-positive T cells (right).

(H-J) Related to IL-22 KO mice described in Fig. 6G. Data were pooled from 2 independent experiments.

(H) Quantification of colon length.

(I) Quantification of clonogenicity capacity of the colon crypts (n = 5-7).

(J) Heatmap with relative mRNA expression of IL-22-target genes of colonic epithelial cells by RT-qPCR.

In all graphs, each point represents an individual animal. Unless otherwise stated, results were analyzed by Student’s t-test. *p < 0.05, **p < 0.01, ***p < 0.001, and ns = not significant.


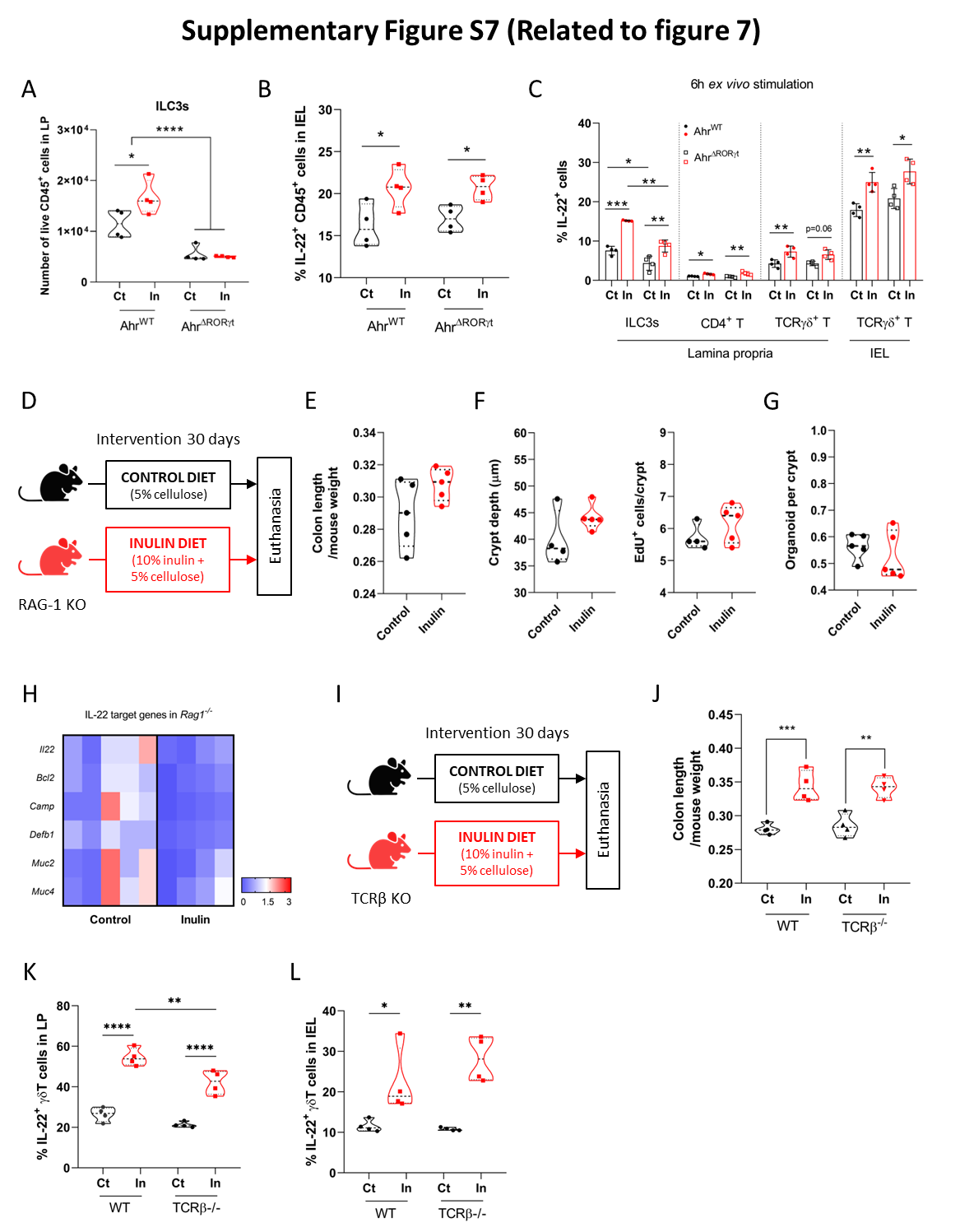


**Supplementary Figure 7 (Related to Figure 7)**

(A) Quantification of ILC3s in lamina propria. Results analyzed by two-way ANOVA (n = 4).

(B) Quantification of the percentage of IL-22-positive CD45+ cells in the intraepithelial lymphocyte population. Results analyzed by two-way ANOVA (n = 4).

(C) Percentage of IL-22 production of each cell type from LP or IEL 6 hours after *ex vivo* culture stimulation. Results analyzed by two-way ANOVA (n = 4).

(D) Schematic of the experimental model with RAG-1 KO mice and different diets (n = 5).

(E) Quantification of colon length.

(F) Quantification of colon crypt depth (left) and number of EdU-positive cells per crypt (right).

(G) Quantification of clonogenicity capacity of colon crypts.

(H) Heatmap with relative mRNA expression of IL-22-target genes of colonic epithelial cells by RT-qPCR.

(I) Experimental model scheme with TCRβ KO mice and different diets (n = 4).

(J) Quantification of colon length.

(K) Quantification of percentage of IL-22-positive γδ T cells in the LP. Results analyzed by one-way ANOVA (n = 4).

(L) Quantification of IL-22-positive γδ T cells in the intraepithelial lymphocyte population. Results analyzed by one-way ANOVA (n = 4).

In all graphs, each point represents an individual animal. Unless otherwise stated, results were analyzed by Student’s t-test. *p < 0.05, **p < 0.01, ***p < 0.001, ***p < 0.001, ****p < 0.0001.
